# Global Methylation Patterns in Idiopathic Pulmonary Fibrosis

**DOI:** 10.1371/journal.pone.0033770

**Published:** 2012-04-10

**Authors:** Einat I. Rabinovich, Maria G. Kapetanaki, Israel Steinfeld, Kevin F. Gibson, Kusum V. Pandit, Guoying Yu, Zohar Yakhini, Naftali Kaminski

**Affiliations:** 1 Dorothy P. and Richard P. Simmons Center for Interstitial Lung Disease, Division of Pulmonary, Allergy and Critical Care Medicine, University of Pittsburgh, School of Medicine, Pittsburgh, Pennsylvania, United States of America; 2 Department of Computer Sciences, Technion – Israel Institute of Technology, Haifa, Israel; 3 Agilent Laboratories, Tel-Aviv, Israel; Helmholtz Zentrum München/Ludwig-Maximilians-University Munich, Germany

## Abstract

**Background:**

Idiopathic Pulmonary Fibrosis (IPF) is characterized by profound changes in the lung phenotype including excessive extracellular matrix deposition, myofibroblast foci, alveolar epithelial cell hyperplasia and extensive remodeling. The role of epigenetic changes in determining the lung phenotype in IPF is unknown. In this study we determine whether IPF lungs exhibit an altered global methylation profile.

**Methodology/Principal Findings:**

Immunoprecipitated methylated DNA from 12 IPF lungs, 10 lung adenocarcinomas and 10 normal histology lungs was hybridized to Agilent human CpG Islands Microarrays and data analysis was performed using BRB-Array Tools and DAVID Bioinformatics Resources software packages. Array results were validated using the EpiTYPER MassARRAY platform for 3 CpG islands. 625 CpG islands were differentially methylated between IPF and control lungs with an estimated False Discovery Rate less than 5%. The genes associated with the differentially methylated CpG islands are involved in regulation of apoptosis, morphogenesis and cellular biosynthetic processes. The expression of three genes (STK17B, STK3 and HIST1H2AH) with hypomethylated promoters was increased in IPF lungs. Comparison of IPF methylation patterns to lung cancer or control samples, revealed that IPF lungs display an intermediate methylation profile, partly similar to lung cancer and partly similar to control with 402 differentially methylated CpG islands overlapping between IPF and cancer. Despite their similarity to cancer, IPF lungs did not exhibit hypomethylation of long interspersed nuclear element 1 (*LINE-1*) retrotransposon while lung cancer samples did, suggesting that the global hypomethylation observed in cancer was not typical of IPF.

**Conclusions/Significance:**

Our results provide evidence that epigenetic changes in IPF are widespread and potentially important. The partial similarity to cancer may signify similar pathogenetic mechanisms while the differences constitute IPF or cancer specific changes. Elucidating the role of these specific changes will potentially allow better understanding of the pathogenesis of IPF.

## Introduction

Idiopathic pulmonary fibrosis (IPF) is a non-neoplastic pulmonary disease, characterized by extracellular matrix deposition, myofibroblasts foci formation and alveolar epithelial cell hyperplasia [1], [2], [3], [4]. The disease is progressive and in most cases unresponsive to corticosteroid and immunosuppressive therapy [5]. Although the exact etiology of the disease is still under investigation, several studies suggest that a combination of genetic and environmental factors may be the cause of IPF [6]. Exposure to wood, metal dust or stone/sand/silica as well as smoking, farming and handling livestock are associated with IPF in several independent studies [7]. A unique feature to the lung phenotype in IPF is the extent to which the lung is altered from normal. Alveolar epithelial cells and fibroblasts exhibit distinct and profound changes in their phenotypes with alveolar epithelial cells undergoing hyperplasia and potentially epithelial mesenchymal transdifferentiation and fibroblasts becoming activated and exhibiting myofibroblast features. Multiple studies demonstrated that the lung phenotype in IPF is dramatically different than that of the healthy lung with globally different patterns of mRNA and microRNA expression [8], [9], [10], [11], [12], [13], [14] and aberrations in multiple pathways such as coagulation [15], apoptosis [16], [17], oxidative stress [18], epithelial mesenchymal transition [19], [20], [21] and developmental pathways [21], [22], [23]. Usually it is assumed that multiple cycles of injury lead to this phenotype, however these injuries do not explain how those profound phenotypic changes are sustained and even progress years after the initial injury. Global epigenetic changes, traditionally defined in the context of heritable changes that are not coded by changes in DNA sequence, have rapidly emerged as a general mechanism by which cellular molecular phenotypes are stably altered during development, cellular differentiation, response to environmental stress and disease pathogenesis [24], [25], [26]. It is well established that nutritional, chemical and physical factors can have a profound effect on gene expression [27]. Not only can they cause mutations in the promoter and coding regions of genes but they can also orchestrate a variety of epigenetic changes [28]. Two of the best described mechanisms of epigenetic control are DNA methylation and chromatin remodeling. DNA methylation typically involves the addition of a methyl group to the 5 position of the cytosine pyrimidine ring of a CpG dinucleotide [29]. Clustered CpGs form CpG islands whose state of methylation is critical for the activity of transposable elements and the transcriptional regulation of genes through direct blockage of transcription factors or chromatin remodeling [30]. Alterations of CpG methylation have been implicated in many diseases where the hypermethylation of the promoter associated CpG islands results in transcriptional silencing [31] while the hypomethylation results in loss of imprinting and transcriptional activation [32]. Aberrant methylation of CpG dinucleotides is a well-recognized epigenetic hallmark of multiple diseases including lung cancers [33], [34], [35], [36]. So far, the extent and role of epigenetic changes has not been studied in IPF.

In this study, we analyze global methylation patterns of IPF using human CpG island microarrays. In addition to compiling a DNA methylation profile that differentiates IPF patients from normal individuals, we compared this profile to that of lung adenocarcinoma patients. Our results reveal an altered DNA methylation pattern in IPF which shows great similarity to the methylation pattern of lung cancer. Our work is the first step in understanding the role of DNA methylation in the pathogenesis of IPF. Furthermore, the similarity of IPF with cancer may reveal common underlying molecular mechanisms and offer therapeutic options for IPF patients adopted from cancer biology [37].

## Methods

### Sample Description

Lung tissue samples were obtained through the University of Pittsburgh Health Sciences Tissue Bank (Pittsburgh, PA). They included 12 frozen lung tissue samples from IPF patients, 10 frozen lung tissue samples from adenocarcinoma patients and 10 histological normal lung samples obtained from the same group of adenocarcinoma patients (**Table 1**). The diagnosis of IPF was based on microscopic findings that were consistent with usual interstitial pneumonia [1], [3]. All adenocarcinoma tumors were obtained from patients staged as T_1b_-T_2b_ N_0_ M_0_ [38]. All cancer patients were smokers and older than IPF patients. The lung samples that were removed from patients with lung cancer contained both adenocarcinoma tissues and normal histology tissues obtained from disease-free margins of the lung. The IPF patients fulfilled the diagnostic criteria of the American Thoracic Society and the European Respiratory Society [1]. Patients consented to the donation of the removed tissue to the tissue bank and the use of the archived tissue was approved by the University of Pittsburgh Institutional Review Board (IRB020123, IRB0506140, IRB0411036). (Pittsburgh, PA).

**Table 1:** Human subjects.

Variable	Control/Cancer	IPF
Number of subjects	10	12
Average age, yr.	71(±12.44)	60(±5.51)
Male/Female	6/4	8/4

### MeDIP and Hybridization to Human CpG Island Microarrays

Genomic DNA was extracted from 10 mg of frozen lung tissues using the DNeasy Blood & Tissue kit (Qiagen Sciences, CA) following the manufacturer’s protocol. Five micrograms of genomic DNA were sonicated (Sonic Dismembrator, Fisher Scientific) to achieve fragment lengths between 300–600 bp. Methylated DNA was immunoprecipitated (IP) using 5-Methylcytidine monoclonal antibody (AbD Serotec, NC) as instructed in the Agilent Microarray Analysis of Methylated DNA Immunoprecipitation protocol (Agilent Technologies, Santa Clara, CA) [39], [40]. Immunoprecipitated DNA and total genomic DNA were labeled with Cy3 and Cy5 respectively, using Agilent Genomic Labeling kit PLUS (Agilent Technologies, Santa Clara, CA) and hybridized to the human CGI oligonucleotide microarrays (Agilent Technologies). The arrays were designed according to the University of California at Santa Cruz (UCSC) genome-browser CpG island list and contained 237,000 probes covering more than 90% of the 27,639 human CpG islands at a density of 1 probe per 100 bp as described in Straussman et al [41] along with quality control assays that assess the platform’s performance. Further validation of Agilent’s MeDIP microarray platform was achieved by Yamashita et al [42]. Following hybridization and washing, the arrays were scanned in an Agilent G2509C microarray scanner and raw data were obtained using Feature Extraction Ver.9.5.3.

### Microarray Data Analysis

For our analysis we only included probes with a hybridization Tm value between 79°C and 93°C as these show higher quality signal [41]. We subsequently divided the probes according to their Tm into 14 groups/bins differing by 1°C. Probe signals in each bin were standardized to have an average of 0 and a standard deviation of 1. To work in a CpG island oriented manner we scored each island for its likelihood to be methylated. For that purpose, each probe was mapped to the genome and the signals of the probes that were mapped to a single CpG island were averaged to obtain the island’s methylation score [41]. The complete microarray data have been deposited in the Gene Expression Omnibus (GSE29895), are MIAME compliant and publicly available.

### MassARRAY EpiTYPER Assay

CpG dinucleotide methylation was quantified by the MassArray EpiTYPER platform (Sequenom Inc, CA) [43]. The EpiTYPER assay is a MALDI TOF mass spectrometry based quantitative method for measuring CpG methylation down to a single dinucleotide resolution. 500 ng of fragmented DNA from each sample was modified by bisulfite treatment. Following PCR with specific primers and Shrimp Alkaline Phosphatase treatment, fragments were ligated to a T7 promoter segment, and then transcribed into RNA. The synthesized RNA was cleaved with RNase A and all cleavage products were analyzed by MassArray in the Genomics and Proteomics Core Laboratory (GPCL, University of Pittsburgh, Pittsburgh, PA) according to the manufacturer’s instructions. Primers were designed using the EpiDesigner Software (http://www.epidesigner.com/index.html) (Table S1 in Supporting Information).

### Quantitative Real-Time Polymerase Chain Reaction (qRT-PCR)

Total RNA was extracted from frozen lung tissue with miRNeasy mini kit (Qiagen Sciences,CA) following the manufacturer’s protocol [44]. 500 ng of the extracted RNA sample was used as a template for the reverse transcriptase reaction. 25 ng of the synthesized cDNA was amplified in a qPCR reaction using TaqMan universal PCR master mix (Applied Biosystems, Foster City,CA) and TaqMan gene expression assays for the following genes: STK17B (assay IDHs00177790_m1), STK3 (assay Hs00169491_m1), HIST1H2AH (assay Hs00544732_s1) and GUSB (assay Hs99999908_m1 ). All assays were done in triplicates and appropriate Non-Transcriptase and Non-Template control reactions were included. GUSB (encoding b-glucoronidase) was used as a housekeeping gene for normalization and the results were analyzed by the ΔΔCT method [45] after averaging the triplicates of each assay. Fold change was calculated by taking the average of all the control samples as the baseline.

### Data Analysis

Differentially methylated CpG islands were identified by analyzing the CpG Island Microarray data with the Class Comparison feature of BRB-ArrayTools 3.7.0 (http://linus.nci.nih.gov/BRB-ArrayTools.html). We controlled for multiple testing by setting the significance level at a False Discovery Rate (FDR) of less than 5% [46]. Data visualization was accomplished using the Genomica [47] and the JavaTreeView software packages. The Student’s *t* test was applied to for the EpiTYPER MassArray and qRT-PCR to test significance of the results. Significance of overlap of differentially methylated islands (DMI) between IPF and Cancer samples and enrichment of DMIs in promoter regions was calculated using the hypergeometric distribution. Pathway analysis was performed using DAVID Bioinformatics Resources 6.7 [48] and IPA Ingenuity Systems (http://www.ingenuity.com).

## Results

The patterns of DNA methylation in lung samples of IPF, cancer patients and controls, were determined using Agilent Human CpG Islands microarrays. Overall, 12 IPF, 10 lung adenocarcinoma and 10 normal histology samples from the same adenocarcinoma patients were included in our study (**Table 1**). The analysis of the microarray data was divided into two parts. In the first part, the IPF or the adenocarcinoma samples were compared to the control samples to compile two separate lists of differentially methylated CpG islands. In the second part, the two lists were compared to assess for differences or similarities between the methylation changes that are associated with each disease.

### IPF Lung Samples Show a Different Methylation Profile when Compared to Normal Histology Lung Samples

**Figure 1:**
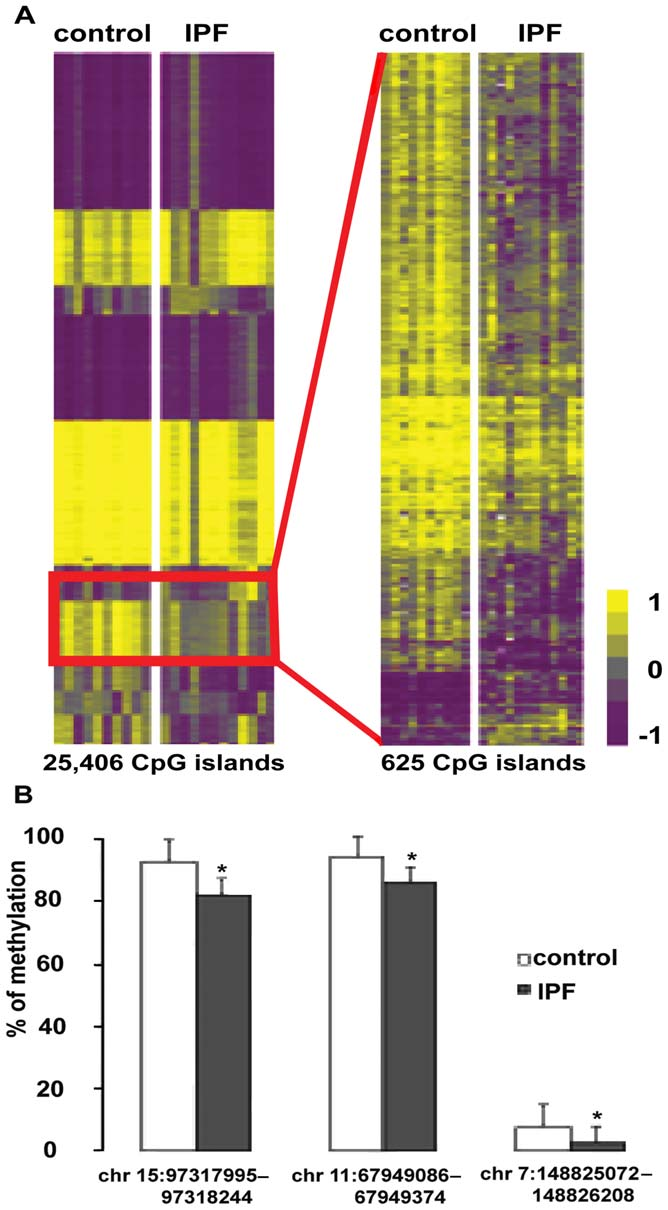
CpG islands are differentially methylated in IPF and control samples (A) Human CpG Island Microarray data: the heatmap on the left is the visual comparison of global methylation profiles between the 10 Control and the 12 IPF samples. The heatmap on the right consists only of the differentially methylated CpG probes highlighted by the red rectangle (*p*-value < 0.05, FDR<5%). Methylated CpG islands are shown in progressively brighter shades of yellow, depending on the fold difference, and hypomethylated CpG islands are shown in progressively brighter shades of purple. Grey stands for no difference between the two sample groups being compared. (B) EpiTYPER confirmation of differentially methylated CpG islands. Bars represent the average methylation of all the samples per study group. The X-axis shows the genomic location of each CpG island and Y-axis shows the percentage of methylation.

The 25,406 out of 27,639 human CpG islands that had an acceptable Tm (see methods) were analyzed using the Class Comparison algorithm from BRB Array Tool software package. 625 CpG islands were found to be differentially methylated in IPF lung tissue samples when compared to control lung tissue samples (**Figure** **1A** **, Table S2** in Supporting information). 91.2% of the 625 differentially methylated CpG islands were located in intronic, exonic or and intergenic areas and only 8.8% in promoters. Considering that 10,923 of the 25,406 (43%) CpG islands in our study localize to promoters, this result indicates that a significantly larger than expected (p < 10^–79^) proportion of changes in methylation, when comparing IPF and control samples, occurs in regions that are not annotated as promoters in the current genome build.

To validate the microarray results, 3 differentially methylated CpG islands showing various degrees of change in their methylation levels were picked and analyzed with the Sequenom’s MassArray EpiTYPER assay. The EpiTYPER assays showed decreased CpG island methylation in the IPF lung samples which was in agreement with the microarray data (**Figure 1B**). All differentially methylated CpG islands were mapped to the genome using the UCSC genome browser [49] and a list of genes that contain CpG islands showing significantly hyper- or hypomethylation in IPF lung samples was compiled (**Table S2** in Supporting Information). A Functional Annotation Clustering of these genes using DAVID Bioinformatics Resources 6.7 revealed that a significant number of them are involved in apoptosis, cell morphogenesis, the regulation of cellular biosynthetic processes and histone acetylation (**Table 2**). The modified Fisher Exact p-Value/EASE Score is calculated to measure gene-enrichment in any given annotation term. It ranges from 0 to 1 with 0 representing perfect enrichment. “Score” stands for Group Enrichment Score, which is calculated using the p-values of the individual members of each Functional Annotation Cluster. The higher the number is the higher the cluster ranks in biological significance [48].

**Table 2:** Functional annotation clustering of differentially methylated CpG islands.

Cluster	Score	Count	p-Value	Genes
regulation ofapoptosis	11.5	23	2.86E-12	COL18A1,OBSCN,PRKCZ,MAEA,WFS1,BCAR1, STK17B,INTS1,ZBTB16,TNFRSF4,STK3,SRC, MCF2L,PROC,AKT1,IGF1R,NOTCH1,IGF2R, BIRC8, BCL6,DNAJB6, ARHGDIA,TERT
negative regulation of cellular biosynthetic process	10.5	19	3.39E-11	EIF2C2,CTBP1,CTBP2,NACC2,EHMT1.JARID2, RBM15B,ZEB2,CBX2,ZBTB16,PRDM16,TNFRSF, HDAC4,ZGPAT,STRA8,BCL6,NCOR2,DNAJB6, SMARCA4
regulation of cell morphogenesis	3.3	5	3.03E-03	PLXNA3,PLXNB2,CDH4,ARHGDIA,MBP
histone acetylation	2.8	4	1.36E-03	KAT2A,BRD1,KAT2B,CREBBP

### Decrease in Promoter CpG Island Methylation is Associated with Increased Gene Expression

**Figure 2:**
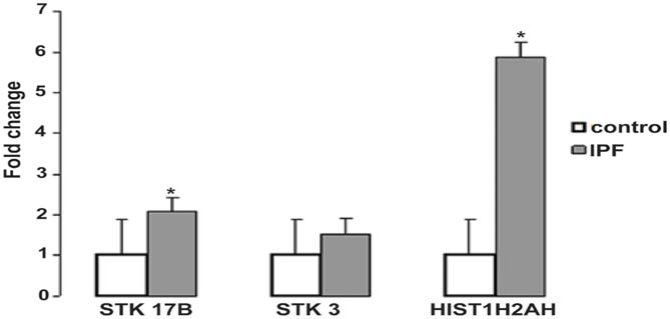
Expression of genes with differentially methylated promoters. qRT-PCR assay on 3 genes with hypomethylated promoter-associated CpG islands showed increase in the expression of the downstream gene. Y-axis shows fold change of detected transcripts in IPF samples when the expression in controls is set to baseline equal to 1. * denotes p-values <0.05. Error bars are based on Standard Deviation.

Typically, the methylation of promoter localized in CpG islands affects gene expression of the downstream genes [50]. All 625 differentially methylated CpG islands were checked for promoter localization and presence of a Trascriptional Start Site (TSS) using the UCSC Genome Browser [49]. 55 CpG islands were mapped in the promoter region of genes that had a well-characterized TSS (**Table 3**). An IPA functional analysis showed that the genes with differentially methylated CpG islands in their promoters are associated with biological processes such as cellular assembly and organization, cellular growth and proliferation, cell morphology, cancer ,cell signaling, gene expression and cell death (**Table S3** in Supporting Information). We analyzed by qRT-PCR three genes localized in differentially regulated regions. Serine/Threonine Kinase 17b (STK17B) and Serine/Threonine Kinase 3 (STK3) are involved in apoptosis while histone cluster 1 H2ah (HIST1H2AH) is essential is nucleosome formation. All three transcripts showed increased levels of expression in IPF samples compared to controls but only STK17B and HIST1H2AH have a p-value <0.05 while in the case of STK3 the p-value is 0.07 (**Figure 2**).

**Table 3:** Differentially methylated promoters between IPF and control samples.

CpG location	Gene	Eponine Predicted TSS	SwitchGear GenomicTSS	p-value	Fold change
chr2:242678973-242680235	LOC728323	chr2.35165-35173	CHR2_P1372	5.70E-06	0.48
chr7:148825072-148826208	ZNF 746	chr7.53295-53308	CHR7_M1039	1.19E-03	0.52
chr19:2653532-2653987	GNG7	chr19.27595-27597	CHR19_M0064,CHR19_P0	1.72E-03	0.53
chr22:37569114-37570371	NPTXR	chr22.39508-39517	CHR22_M0327	3.77E-05	0.56
chr12:119290634-119291409	MSI1	chr12.12663-12672	CHR12_M0815	4.79E-05	0.58
chr17:53515753-53516493	DYNLL2	chr17.23937	CHR17_P0699	1.10E-04	0.58
chr8:99906637-99907112	STK3	chr8.55187	CHR8_M0508	3.76E-04	0.58
chr19:52614063-52614589	MEIS3		CHR19_M0732	3.60E-04	0.59
chr4:75122817-75123453	CXCL3		CHR4_M0336	3.54E-04	0.59
chrY:2537106-2537697	NCRNA00103	chrY.61288	CHRY_P0007	2.22E-04	0.59
chr9:126945496-126945768	SCAI		CHR9_M0684	3.13E-04	0.59
chr13:31318630-31319274	EEF1DP3	chr13.13604-13608	CHR13_P0091	2.02E-04	0.6
chr1:46906261-46906982	ATPAF1	chr1.2564-2568		7.34E-04	0.6
chr1:76312735-76313241	ST6GALNAC3	chr1.2978,2979	CHR1_P0726_R1	1.10E-04	0.61
chr6:27222201-27223160	HIST1H2AH	chr6.48519	CHR6_M0196,CHR6_P0200	6.12E-04	0.61
chr20:46971570-46972079	ARFGEF2	chr20.36249-36250	CHR20_P0396	1.65E-04	0.62
chr20:390530-391317	TBC1D20	chr20.35209-35211	CHR20_M0004	3.33E-04	0.62
chr3:20056462-20057430	KAT2B	chr3.40742-40745	CHR3_P0121	1.15E-04	0.63
chr8:1908966-1910279	KBTBD11	chr8.53949-53953	CHR8_P0014,CHR8_M0	3.49E-04	0.63
chr9:21549133-21549816	LOC554202	chr9.56427	CHR9_M0115	9.90E-04	0.63
chr3:44493842-44494372	ZNF445	chr3.41072-41077	CHR3_M0205	3.93E-05	0.64
chr7:16427303-16427790	ISPD	chr7.51105		4.83E-04	0.64
chr7:4889233-4890102	RADIL	chr7.50913-50914	CHR7_M0042	4.83E-04	0.64
chr10:73203097-73203498	C10orf54	chr10.6463-6464		1.31E-03	0.64
chr17:35036840-35037445	PPP1R1B	chr17.23254		9.02E-04	0.64
chr2:37237405-37237906	EIF2AK2		CHR2_M0214	7.71E-04	0.64
chr2:196743834-196744628	STK17B		CHR2_M1078	4.38E-04	0.65
chr7:105539339-105540384	SYPL1	chr7.52701-52704	CHR7_M0755	7.45E-04	0.65
chr5:177949164-177950276	COL23A1		CHR5_M0978	5.10E-04	0.66
chr9:138236486-138236814	LHX3		CHR9_M0813	9.64E-05	0.66
chr6:38715755-38716126	BTBD9	chr6.48830		6.01E-04	0.67
chr22:37481403-37482422	SUN2	chr22.39495-39505	CHR22_M0325.1	1.15E-03	9.68
chr17:77421925-77423424	ARHGDIA	chr17.24948-24955	CHR17_M1049	1.00E-03	0.68
chr1:219026639-219027226	MOSC1	chr1.4565	CHR1_P1706_R1	5.74E-04	0.69
chr16:23429074-23429400	GGA2	chr16.19779	CHR16_M0269_R1	2.76E-04	0.71
chr6:35996457-35997038	SRPK1		CHR6_M0389	8.45E-04	0.71
chr4:77391611-77392084	FAM47D	chr4.44416-44418	CHR4_P0385	2.44E-04	0.72
chr3:52714577-52715466	GLT8D1-SPCS1	chr3.41472-41477	CHR3_M0331,CHR3_P0373	3.65E-04	0.72
chr19:40015371-40015792	LOC400685	chr19.29348	CHR19_M0487	1.08E-03	1.36
chr1:154808723-154809126	IQGAP3		CHR1_M1183_R1	3.07E-04	1.37
chr11:64645712-64646507	FAU-MRPL49	chr11.9471-9474	CHR11_M0531_R1	8.27E-04	1.37
chr22:36575287-36575627	EIF3L		CHR22_P0282	1.21E-03	1.47
chr19:62972738-62973298	ZNF 586		CHR19_P0955.3	1.01E-03	1.49
chr2:10747175-10747692	NOL10		CHR2_M0064, CHR2_P0054	8.54E-04	1.51
chr22:29886124-29886466	RNF185		CHR22_P0227	2.63E-04	1.53
chr4:17225273-17225604	MED28		CHR4_P0145	1.32E-03	1.53
chr4:191142227-191143118	TUBB4Q	chr4.45402		9.16E-05	1.53
chr17:24206105-24206445	ERAL1		CHR17_P0310	6.41E-04	1.56
chr17:34610012-34610471	RPL19		CHR17_P0434	3.92E-04	1.56
chr3:50357893-50358314	ZMYND10	chr3.41343	CHR3_M0300	2.53E-04	1.56
chr1:40008354-40009777	BMP8B	chr1.2303		3.91E-04	1.61
chr1:68288824-68289061	DIRAS3	chr1.2955		1.58E-04	1.63
chr3:159844871-159845169	GFM1		CHR3_P0882	1.28E-03	1.66
chr3:139211681-139211893	CLDN18		CHR3_P0776	4.00E-06	1.69
chr5:169592408-169592807	C5orf58		CHR5_P0975	1.70E-06	1.81

### The Methylation Profiles of IPF and Adenocarcinoma Lung Samples Overlap

**Figure 3:**
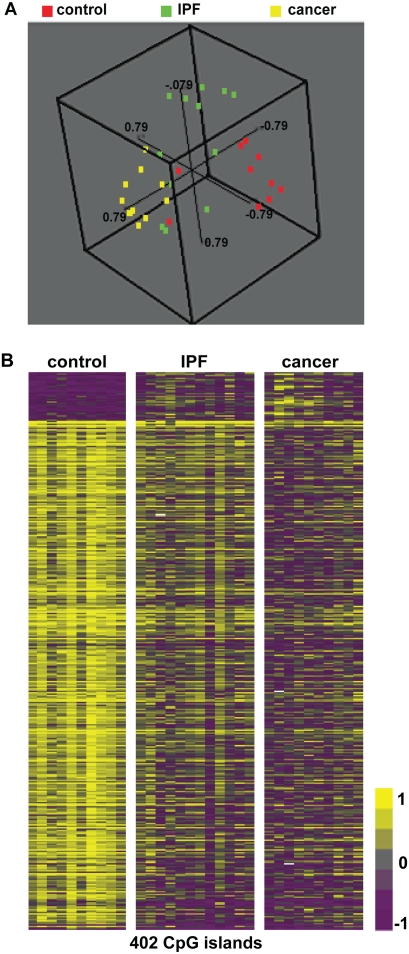
Comparison of IPF and Adenocarcinoma to control samples. (A) 3-D plot representation of the results after Principal Component Analysis of all 3 sample groups. Each color-coded dot represents a sample (red-control, green-IPF and blue-cancer) and each sample is positioned in the 3-D space according to its similarity or difference to the others. (B) Comparison of differentially methylated CpG islands that overlap between IPF and Lung Cancer. The heatmap consists of 402 differentially methylated CpG islands that are found to overlap between IPF and Lung Cancer. High methylation levels of a CpG islands are shown in yellow while low methylation levels of methylation are shown in purple. Grey stands for no difference between the two groups being compared.

To determine the similarity of IPF samples to lung cancer we performed Principal Component Analysis (PCA) and Class Comparison using BRB-Array Tools on microarray data from 12 IPF patients and the 10 lung cancer patients (normal histology and adenocarcinoma samples included). PCA analysis demonstrated that IPF samples were positioned between the control and cancer samples suggesting that IPF samples had a methylation profile with partial similarity to both groups (**Figure 3A**). It is worth mentioning that IPF samples were more similar to cancer than control samples despite the fact that cancer and control tissue were obtained in pairs from the same patient. This observation may suggest that the majority of differences between IPF and controls may be related to differential environmental exposures or smoking effect because these differences persisted in the comparison of cancer to control despite the fact they came from the same subject. Class comparison analysis revealed that 2428 CpG islands were differentially methylated between cancer samples and normal histology controls. When compared to the 625 that are differentially methylated between IPF and Controls, 402 CpG islands overlapped. In other words, 65% of the CpG islands that have an altered methylation pattern in IPF lung samples are also modified in lung cancer samples (**Figure 3B** and **Table S4** in Supporting Information). This overlap is highly significant, as the probability of such an overlap to occur in random is very low (p<10^−256^). 45% of the 402 overlapped CpG islands are located in intronic and intergenic areas, 6% in promoters and 49% in exons.

**Figure 4:**
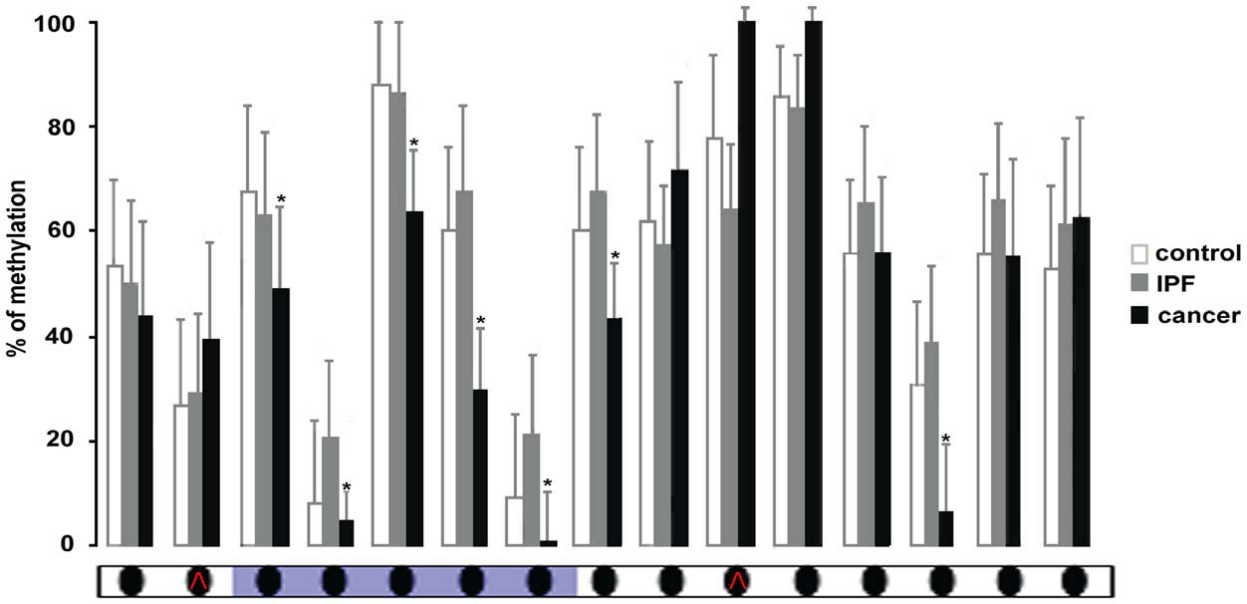
Methylation profile of LINE-1 retrotransposon. Global methylation levels of the LINE-1 retrotransposons were defined using the EpiTYPER mass array assay. Each bar represents the methylation in one of the 15 CpG dinucleotides or CpG units that span the LINE-1 sequence. Methylation levels are calculated as the average of all samples in each group (Control, IPF, Cancer) and standard error bars are included. The X axis shows the CpG dinucleotide or the CpG unit and the Y axis shows the percentage of methylation. The LINE-1 promoter is indicated in purple. The red arrowheads indicate units of 2-3 CpG dinucleotides.

To determine whether similar methylation patterns in IPF and cancer result from a global change in methylation we assessed LINE-1 methylation. LINE-1 retrotransposons are abundantly and equally distributed across the genome and their methylation pattern is often used as an indicator of global methylation levels [51]. The methylation status of LINE-1 (GenBank: X58075.1) was defined in all three study groups (IPF, Cancer and Control) using the EpiTYPER MassArray assay. The PCR primers were designed to encompass the 15 CpG sites or units including the possible intrinsic LINE-1 promoter (**Table S1** in Supporting Information). Although LINE-1 elements were found to be hypomethylated in the adenocarcinoma samples no significant change of the methylation levels was detected the in IPF samples (**Figure** **4**) suggesting that methylation changes in IPF were specific to regions.

## Discussion

In this study, we used human CpG island microarrays to identify the differentially methylated CpG islands in the lung tissue of IPF patients. Our results indicate that the CpG island methylation profile of the IPF lung samples is very different from that of control samples and greatly overlaps with methylation changes observed in lung adenocarcinoma samples. Despite the observed similarity in CpG methylation between IPF and lung cancer, the lack of LINE-1 methylation in IPF suggests a more specific DNA methylation, which is confined to certain regions of the genome.

One of the most impressive results of our study is the extent of differentially methylated regions in the IPF lungs. Interestingly the majority of the differentially methylated CpG islands rest in promoter-distal sites or intragenic regions and only 8.8% of them are localized in gene promoters. Whereas the methylation status of promoter associated CpG islands can directly affect transcription, the role of the CpG methylation outside the immediate promoter region remains somewhat unclear. It is proposed that methylation of CpG island shores outside the promoter could also control transcription of downstream genes [52] or lead to histone modifications [53]. Methylation changes that occur in intragenic regions could impact RNA splicing [54]. In addition, methylation changes may affect the expression of non-coding RNAs [55] and thus indirectly affect global changes in gene expression. The biological impact of modest changes in the degree of CpG methylation is in fact unpredictable. As an example in the case of prostate cancer, a gradual increase in methylation from 12.6% to 19.3% or 21.8% signified a transition from a benign state to a localized or metastatic cancer, respectively [56]. However, regardless of the direct downstream effects, the extent of the methylation changes we found, supports previous observations about the degree and profundity of molecular changes in the IPF lung [8], [9], [10], [11], [12], [13], [14].

Naturally, the motivation to assemble methylation profiles is to find the underlying mechanisms that drive changes in gene expression. The detailed characterization of each one of the differentially methylated CpG islands in IPF patients is beyond the scope of this study. Globally, genes with differentially methylated CpG islands in their promoters were involved in biological processes such as cellular assembly and organization, cellular growth and proliferation, cell morphology, cancer, cell signaling, gene expression and cell death. All of these processes could be implicated in IPF pathogenesis. In our validation we focused on genes with differentially methylated promoters. We selected the Serine/Threonine Kinase 17b (STK17B) and Serine/Threonine Kinase 3 (STK3) because of their role in apoptosis [57] and the histone cluster 1 H2ah (HIST1H2AH) because of the recent interest in histone modifications in fibrosis[58]. STK17B and HIST1H2AH were significantly up-regulated in our IPF samples which is in agreement with the hypo-methylated state of their promoter associated CpG islands. Interestingly, the majority of the differentially methylated islands that were within or close to known genes were outside promoter regions. Some of these methylation changes were in genes that were previously reported to be increased in IPF such as COL18A1 [11], genes that are implicated in myofibroblast differentiation such as NOTCH1 [59] or markers of progressive IPF like SMARCA4 [60]. In addition, the promoter of CXCL3, a gene which is found to be up-regulated in the lung of bleomycin treated mice [61], was also hypomethylated in our IPF samples. When we looked for the overlap of differentially expressed genes in IPF in our previously published gene expression datasets [13], [14] we found that there were 46 genes that had both differentially methylated gene related CpG islands and gene expression changes. While a detailed analysis of methylation and expression changes in the same tissue would be better suited to address the correlation of methylation and gene expression changes our findings suggest that at least some of the methylation changes that we observed do have an effect on lung gene expression and thus may contribute to the lung phenotype in IPF.

A remarkable finding of our study is the similarity in DNA methylation patterns between IPF and lung adenocarcinoma. Recently, Vancheri *et al* compared IPF to cancer and described the pathogenic similarities between the two diseases. More specifically, they referred to common genetic and epigenetic alterations, uncontrolled proliferation, tissue invasion and perturbation of signal transduction pathways [37]. The similarity between cancer and IPF spreads to microRNA expression such as in the case of let-7d and hsa-miR-21, which are found to be down-regulated or up-regulated respectively in both diseases [8], [9]. All of these observations are in accord with published studies reporting high incidence of cancer in IPF patients when compared to healthy individuals [62], [63]. DNA hypomethylation is a hallmark of cancer [64] and in many types of cancer including lung carcinomas it is accompanied with lower levels of methylation in repetitive DNA elements [34], [65]. While the similarity in the differentially methylated CpG islands suggests common epigenetic mechanisms between IPF and cancer, our analysis of LINE-1 methylation indicates that this similarity is limited. LINE-1 repeats comprise about 20% of the human genome [66]. LINE-1 elements are usually methylated in somatic tissues but they are often hypomethylated in tumors [65], [67] resulting in increased mobility, which in turn leads to gene disruptions [68] and chromosomal instability [69]. While LINE-1 retrotransposons were hypomethylated in our cancer samples they were not in IPF samples leading to the conclusion that CpG island methylation changes in IPF are somewhat parallel to cancer but are not as extensive and do not involve global changes in LINE-1 methylation. This suggests that despite the similarities between the DNA methylation profiles of IPF and cancer, there are different mechanisms that cause and sustain these changes.

One of the major concerns in our global profiling approach is tissue heterogeneity. The IPF lungs contain mixed areas of normal tissue, myofibroblast foci and honeycombing [1]. The IPF lung is also highly variable in its cellular content as it contains normal cells like epithelial, endothelial cells and fibroblasts as well as abnormal ones like hyperplastic type II alveolar epithelial cells, myofibroblasts, potentially altered endothelial cells and varying degrees of inflammatory cells. Thus it is possible that the signal we obtained is only an under-estimation of the real epigenomic changes caused by an admixture of normal and abnormal regions, microenvironments and cell types. Naturally, it is impossible based on our analysis to determine whether the observed DNA methylation changes are cell type specific. In this context, our strategy of averaging signals across an island could also lead to loss of information and underestimation of epigenetic changes. However, we chose this approach because although it is less sensitive, we felt it provided us with global results, reduced the need to deal with probe variability and provided a good approximation of differentially methylated CpG islands. In the future it may make sense to refine both the measurement approach and data analysis to obtain more detailed results. The heterogeneity of our samples as well as the different methodologies used to identify the differences in CpG methylation could also explain the absence of PTGER2 and Thy-1 from our list of significantly methylated genes. The promoters of PTGER2[70] and Thy-1[71] were found to be hypermethylated in fibrotic lung fibroblasts and fibrotic tissue from IPF patients resulting in low levels of the coded proteins. In fact Thy-1 it is shown that the downregulation occurs only in areas of dense fibrosis and fibrotic foci while the rest of the tissue remains unaffected [70], [71]. However, the demonstration of significant global methylation changes despite the limitations of our methods, may be indicative of the importance of epigenomic regulation in IPF and lead to many more detailed discoveries and insights.

To the best of our knowledge our study is the first one to describe global DNA methylation changes in IPF lungs. Taken together with the extensive changes in gene histology, gene expression and microRNA profiles our results highlight the profundity and complexity of events underlying the phenotypic changes in IPF and to some extent suggest that interfering with one pathway may not be sufficient to reverse these changes. The differentially methylated CpG islands we identified should be further studied as their regulation could provide insights about how genotype and the environment interact to determine the lung phenotype in IPF. Based on our results, we believe that epigenetic modifications play a key role in the pathogenesis of IPF and thus could serve as disease biomarkers and therapeutic targets.

## Supporting Information

Table S1 **The sequence of EpiTYPER MassArray primers.**
(DOC)

Table S2 **Differentially methylated CpG islands distinguishing IPF from controls.**
(DOC)

Table S3 **Functional Analysis of the 55 Differentialy methylated promoters in IPF.**
(DOC)

Table S4 **Differentially methylated CpG islands overlapping between IPF and cancer.**
(DOC)
