# Supplementary material for: Global Methylation Patterns in Idiopathic Pulmonary Fibrosis
Source: PLoS One. 2012 Apr 10;7(4):e33770. doi: 10.1371/journal.pone.0033770 (PMC3323629; doi:10.1371/journal.pone.0033770)
Supplement: Table S1 — The sequence of EpiTYPER MassArray primers. (DOC) [file pone.0033770.s001.doc]

**Table S1. The sequence of EpiTYPER MassArray primers**

| **CpG island's location** | **Primer** | **Sequence** | **Length (bp)** | **Product size/ assayed CGs (bp/n)** | **Tm (°C)** |
| --- | --- | --- | --- | --- | --- |
| chr11:67949086-67949374 | Forward 10F | GTGTTTAGGAGTTTTGGTTTTTTTG | 25+10 | 440/21 | 62 |
|  | Reverse T7R | CCTTCCAACCCTTAAAACTTTCTAC | 25+31 |  |  |
| chr15:97068317-97068541 | Forward 10F | GGATTTGGAGTTAGAGAATATGGAGA | 26+10 | 357/23 | 60 |
|  | Reverse T7R | CAACAAAAACAATTCTAAAAATCCAC | 26+31 |  |  |
| chr7:148825072-148826208 | Forward 10F | TTATTGAAAGGGTTTTTTTTATGGG | 25+10 | 237/18 | 60 |
|  | Reverse T7R | TTCCATTTCAAACACAAATAAACAT | 25+31 |  |  |
| LINE-1 retrotransposon | Forward 10F | GGGTGATTTTTGTATTTTTATTTGAG | 26+10 | 500/19 | 60 |
|  | Reverse T7R | TTATCTATACCCTACCCCCAAAAAT | 26+31 |  |  |
