# Supplementary material for: Global Methylation Patterns in Idiopathic Pulmonary Fibrosis
Source: PLoS One. 2012 Apr 10;7(4):e33770. doi: 10.1371/journal.pone.0033770 (PMC3323629; doi:10.1371/journal.pone.0033770)
Supplement: Table S2 — Differentially methylated CpG islands distinguishing IPF from controls. (DOC) [file pone.0033770.s002.doc]

**Table S2. Differentially methylated CpG islands distinguishing IPF from controls**

| **CpG island location** | **Gene Symbol** | **Locus** | **DNA region** | **p-value** | **Fold-change** |
| --- | --- | --- | --- | --- | --- |
| **hypomethylated CpG islands** | | | | | |
| chr1:1016240-1016488 | C1orf159 | p36.33 | intron | 1.33E-04 | 0.64 |
| chr1:10630397-10630727 | CASZ1 | p36.22 | exon1 | 9.27E-04 | 0.63 |
| chr1:1136571-1136777 | TNFRSF4 | p36.33 | exon1 | 9.57E-04 | 0.64 |
| chr1:1151822-1152144 | SDF4 | p36.33 | intron | 7.02E-04 | 0.66 |
| chr1:1445165-1445755 | ATAD3A | p36.33 | exon-intron | 4.62E-04 | 0.58 |
| chr1:144802491-144802782 | CpG 23 | q21.1 | intergenic | 1.98E-04 | 0.58 |
| chr1:145846762-145847106 | GJA8 | q21.1 | exon1 | 2.55E-04 | 0.7 |
| chr1:146184780-146185539 | NBPF11 | q21.1 | intron | 1.43E-05 | 0.65 |
| chr1:147120620-147122385 | CpG 202 | q21.1 | intergenic | 1.06E-03 | 0.58 |
| chr1:151928827-151929063 | NPR1 | q21.3 | exon-intron | 1.26E-04 | 0.69 |
| chr1:154808723-154809126 | IQGAP3 | q23.1 | promoter-exon1-intron | 3.07E-04 | 0.72 |
| chr1:15926203-15926562 | PLEKHM2 | p36.21 | exon | 3.00E-07 | 0.5 |
| chr1:15930884-15931092 | PLEKHM2 | p36.21 | exon-intron | 1.57E-05 | 0.59 |
| chr1:16347698-16347911 | EPHA2 | p36.13 | exon | 8.93E-04 | 0.7 |
| chr1:1675233-1675831 | NADK | p36.33 | exon, intron | 3.89E-04 | 0.55 |
| chr1:16924078-16924478 | CpG 38 | p36.13 | intergenic | 5.38E-04 | 0.64 |
| chr1:19053532-19054056 | TAS1R2 | p36.13 | exon | 1.33E-05 | 0.62 |
| chr1:19082213-19082501 | ALDH4A1 | p36.13 | exon-intron-exon | 2.01E-05 | 0.58 |
| chr1:2056227-2056526 | PRKCZ | p36.33 | intron | 8.60E-06 | 0.61 |
| chr1:208923682-208923885 | KCNH1 | q32.2 | exon | 3.29E-04 | 0.64 |
| chr1:219026639-219027226 | MOSC1 | q41 | promoter | 5.74E-04 | 1.45 |
| chr1:22328610-22328812 | WNT4 | p36.12 | exon-intron | 1.11E-04 | 0.66 |
| chr1:224141773-224142303 | LEFTY1 | q42. 12 | exon-intron-exon | 2.50E-04 | 1.79 |
| chr1:226528508-226528762 | OBSCN | q42.13 | exon | 6.34E-04 | 0.66 |
| chr1:226540458-226540666 | OBSCN | q42.13 | exon | 2.35E-05 | 0.58 |
| chr1:226614700-226614953 | OBSCN | q42.13 | exon-intron | 5.70E-04 | 0.68 |
| chr1:2294019-2294236 | MORN1 | p36.33 | intron | 6.00E-07 | 0.62 |
| chr1:242080425-242081215 | CpG 70 | q44 | intergenic | 9.18E-04 | 1.36 |
| chr1:2429986-2430338 | PANK4 | p36.32 | exon | 2.85E-04 | 0.65 |
| chr1:243915162-243915726 | KIF26B | q44 | intron-exon | 4.84E-05 | 0.61 |
| chr1:2441077-2441411 | PANK4 | p36.32 | exon-intron | 1.12E-03 | 0.69 |
| chr1:2442655-2442867 | PANK4 | p36.32 | intron | 1.33E-04 | 0.66 |
| chr1:2527531-2527746 | MMEL1 | p36.32 | exon-intron | 1.33E-03 | 0.69 |
| chr1:28157607-28157891 | SMPDL3B | p35.3 | exon | 9.92E-04 | 0.7 |
| chr1:2908225-2908613 | CpG 34 | p36.32 | intergenic | 9.50E-06 | 0.59 |
| chr1:3061759-3062099 | PRDM16 | p36.32 | intron | 1.30E-06 | 0.59 |
| chr1:3174406-3174684 | PRDM16 | p36.32 | intron | 8.70E-06 | 0.62 |
| chr1:33863192-33863441 | CSMD2 | p35.1 | exon | 1.05E-04 | 0.59 |
| chr1:3501569-3501978 | MEGF6 | p36.32 | intron-exon-intron | 5.40E-06 | 0.62 |
| chr1:40008354-40009777 | BMP8B | p34.2 | promoter | 3.91E-04 | 0.62 |
| chr1:43125372-43125581 | CpG 15 | p34.2 | intergenic | 5.10E-04 | 0.69 |
| chr1:43126534-43126757 | CpG 17 | p34.2 | intergenic | 1.13E-04 | 0.63 |
| chr1:46906261-46906982 | ATPAF1 | p33 | promoter | 7.34E-04 | 1.65 |
| chr1:5716144-5716371 | CpG 16 | p36.31 | intergenic | 1.09E-04 | 0.61 |
| chr1:5859744-5859979 | NPHP4 | p36.31 | exon | 1.27E-04 | 0.68 |
| chr1:6575984-6576261 | KLHL21 | p36.31 | exon | 2.70E-04 | 0.62 |
| chr1:68288824-68289061 | DIRAS3 | p31.3 | promoter-exon1 | 1.58E-04 | 0.61 |
| chr1:76312735-76313241 | ST6GALNAC3 | p31.1 | promoter-exon1 | 1.10E-04 | 1.62 |
| chr1:7645987-7647762 | GAMTA1 | p36.23 | exon1 | 2.95E-05 | 0.66 |
| chr1:7924995-7925286 | CpG 31 | p36.23 | intergenic | 3.18E-04 | 0.65 |
| chr1:8308482-8308755 | SLC45A1 | p36.23 | exon-intron | 2.80E-06 | 0.58 |
| chr1:8685592-8686136 | RERE | p36.23 | intron | 1.84E-05 | 1.69 |
| chr1:9247274-9247603 | H6PD | p36.22 | exon1 | 2.50E-05 | 0.62 |
| chr1:9713745-9714073 | CLSTN1 | p36.22 | intron-exon | 1.69E-05 | 0.63 |
| chr10:102474190-102474466 | CpG 30 | q24.31 | intergenic | 7.15E-04 | 1.6 |
| chr10:126676541-126676882 | CTBP2 | q26.13 | exon-intron | 1.10E-04 | 0.61 |
| chr10:131576355-131576585 | EBF3 | q26.3 | intron | 6.61E-04 | 0.61 |
| chr10:133601389-133601624 | PPP2R2D | q26.3 | intron | 6.60E-04 | 0.67 |
| chr10:133866033-133866334 | DPYSL4 | q26.3 | intron-exon | 6.25E-04 | 0.6 |
| chr10:134209523-134209770 | INPP5A) | q26.3 | intron | 1.33E-05 | 0.57 |
| chr10:134431719-134432069 | INPP5A | q26.3 | intron | 1.14E-03 | 0.61 |
| chr10:134455574-134456378 | CpG 64 | q26.3 | intergenic | 1.05E-03 | 0.65 |
| chr10:134474044-134474265 | C10orf92 | q26.3 | intron | 5.89E-04 | 0.63 |
| chr10:134522359-134522594 | CpG16 | q26.3 | intergenic | 1.11E-04 | 0.66 |
| chr10:134671723-134671927 | CpG 16 | q26.3 | intergenic | 1.25E-04 | 0.7 |
| chr10:134676120-134676833 | CpG 56 | q26.3 | intergenic | 6.27E-05 | 0.61 |
| chr10:134870608-134870922 | KNDC1 | q26.3 | exon-intron | 8.52E-05 | 0.7 |
| chr10:13556171-13556753 | BEND7 | p13 | intron | 8.21E-04 | 0.57 |
| chr10:1506979-1507313 | ADARB2 | p15.3 | intron | 4.99E-05 | 0.64 |
| chr10:15294604-15294858 | FAM171A1 | p13 | exon1 | 1.03E-03 | 0.58 |
| chr10:2805003-2805469 | CpG 33 | p15.3 | intergenic | 1.84E-04 | 0.64 |
| chr10:35543814-35544027 | CpG 23 | p11.23 | intergenic | 5.50E-04 | 0.66 |
| chr10:38009142-38010356 | CpG 82 | p11.21 | intergenic | 2.86E-04 | 0.65 |
| chr10:515719-516041 | DIP2C | p15.3 | intron | 2.74E-04 | 0.56 |
| chr10:70678278-70678484 | HKDC1 | q21.3 | exon | 1.97E-04 | 0.64 |
| chr10:73203097-73203498 | CDH23 | q22.1 | intron | 1.31E-03 | 1.54 |
| chr10:99319950-99320194 | UBTD1 | q24.1 | exon | 8.11E-04 | 0.68 |
| chr11:113458830-113459049 | ZBTB16 | q23.2 | intron | 1.50E-06 | 0.55 |
| chr11:114529817-114530154 | CpG 32 | q23.2 | intergenic | 7.54E-04 | 0.64 |
| chr11:1254585-1255052 | TOLLIP | p15.5 | exon-intron | 5.07E-04 | 0.61 |
| chr11:1387515-1388410 | BRSK2 | p15.5 | intron | 3.84E-04 | 0.64 |
| chr11:2774563-2774957 | KCNQ1 | p15.5 | intron | 2.19E-04 | 0.65 |
| chr11:3210815-3211052 | CpG 17 | p15.4 | intergenic | 8.52E-05 | 0.63 |
| chr11:43500411-43500662 | CpG 20 | p11.2 | intergenic | 8.64E-04 | 0.65 |
| chr11:462781-463065 | PTDSS2 | p15.5 | intron | 8.50E-06 | 0.59 |
| chr11:601691-601943 | PHRF1 | p15.5 | exon1 | 3.12E-04 | 0.65 |
| chr11:64645712-64646507 | FAU-MRPL49 | q13.1 | Divergent promoter | 8.27E-04 | 0.72 |
| chr11:64928945-64929172 | FRMD8 | q13.11 | exon | 5.19E-04 | 0.64 |
| chr11:67189331-67189627 | ALDH3B2 | q13.2 | exon | 9.00E-07 | 0.54 |
| chr11:67935494-67935738 | LRP5 | q13.2 | exon | 2.51E-05 | 0.69 |
| chr11:67937662-67937865 | LRP5 | q13.2 | exon | 4.66E-05 | 0.63 |
| chr11:67949086-67949374 | LRP5 | q13.2 | exon | 4.88E-05 | 0.62 |
| chr11:69177991-69178333 | CCND1 | q13.2 | exon | 2.72E-04 | 0.66 |
| chr11:69334289-69334654 | FGF3 | q13.3 | exon | 4.69E-04 | 0.72 |
| chr11:69934927-69935204 | CTTN | q13.3 | intron | 7.72E-04 | 0.56 |
| chr12:119018935-119019400 | RAB35 | q24.23 | exon1 | 2.52E-04 | 0.67 |
| chr12:119021023-119021334 | RAB35 | q24.23 | exon | 1.52E+04 | 0.65 |
| chr12:119290634-119291409 | MSI1 | q24.31 | promoter-exon1-intron | 4.79E-05 | 1.72 |
| chr12:120739290-120739877 | SETD1B | q24.31 | exon-intron | 2.97E-04 | 0.71 |
| chr12:123354512-123354837 | FAM101A | q24.31 | intron | 3.48E-04 | 0.64 |
| chr12:123374925-123375129 | NCOR2 | q24.31 | exon1 | 2.01E-04 | 0.58 |
| chr12:123789473-123789703 | CpG 18 | q24.31 | intergenic | 5.92E-04 | 0.57 |
| chr12:130189150-130189496 | GPR133 | q24.33 | intron | 2.86E-04 | 0.63 |
| chr12:13044086-13044351 | HEBP1 | p13.1 | intron-exon | 4.33E-04 | 1.6 |
| chr12:130509472-130509714 | CpG 20 | q24.33 | intergenic | 1.64E-04 | 0.65 |
| chr12:130859396-130859769 | CpG 36 | q24.33 | intergenic | 8.67E-04 | 0.7 |
| chr12:131256292-131256524 | GALNT9 | q24.33 | exon1 | 2.28E-05 | 0.63 |
| chr12:131347484-131347753 | GALNT9 | q24.33 | Exon-intron | 9.20E-06 | 0.54 |
| chr12:131355831-131356429 | GALNT9 | q24.33 | intron | 3.12E-04 | 0.67 |
| chr12:131562753-131562976 | CpG 21 | q24.33 | intergenic | 1.25E-03 | 0.55 |
| chr12:131650377-131650707 | FBRSL1 | q24.33 | intron | 8.05E-05 | 0.65 |
| chr12:6257915-6258429 | CpG 64 | p13.31 | intergenic | 5.08E-04 | 1.42 |
| chr13:109962285-109962548 | COL4A2 | q34 | exon | 2.83E-04 | 0.71 |
| chr13:110099317-110099594 | CARS2 | q34 | intron | 2.82E-04 | 0.63 |
| chr13:110129167-110129374 | CARS2 | q34 | intron | 1.83E-04 | 0.61 |
| chr13:111896576-111897222 | CpG 53 | q34 | intergenic | 3.50E-04 | 0.65 |
| chr13:112155709-112156054 | CpG 26 | q34 | intergenic | 9.28E-04 | 0.65 |
| chr13:112488234-112489058 | ATP11A | q34 | intron | 4.28E-04 | 0.58 |
| chr13:112522081-112522353 | ATP11A | q34 | intron-exon-intron | 8.99E-04 | 0.66 |
| chr13:112534836-112535348 | ATP11A | q34 | intron-exon | 3.53E-04 | 0.66 |
| chr13:112545297-112545662 | ATP11A | q34 | intron | 2.55E-04 | 0.62 |
| chr13:112732526-112732992 | MCF2L | q34 | intron | 9.00E-04 | 0.65 |
| chr13:113023894-113024140 | LAMP1 | q34 | exon-intron | 1.86E-04 | 0.61 |
| chr13:113133424-113133683 | ADPRHL1 | q34 | intron | 2.23E-04 | 0.54 |
| chr13:113246319-113246553 | TMCO3 | q34 | intron | 1.00E-05 | 0.55 |
| chr13:113338025-113338263 | TFDP1 | q34 | intron | 2.70E-04 | 0.65 |
| chr13:113576152-113576502 | GAS6 | q34 | intron-exon-intron | 1.23E-03 | 0.67 |
| chr13:113615752-113616114 | FAM70B | q34 | exon-intron | 7.30E-05 | 0.65 |
| chr13:113804437-113804639 | RASA3 | q34 | intron | 6.10E-06 | 0.57 |
| chr13:113897143-113897632 | RASA3 | q34 | intron | 1.29E-03 | 0.68 |
| chr13:31318630-31319274 | EEF1DP3 | p13.1 | promoter | 2.02E-04 | 1.66 |
| chr13:43441782-43442018 | CpG 17 | q14.11 | intergenic | 8.27E-04 | 0.65 |
| chr14:100418138-100418451 | RTL1 | q32.31 | exon | 1.16E-05 | 0.57 |
| chr14:103234804-103235031 | KLC1 | q32.33 | exon | 5.74E-04 | 0.64 |
| chr14:103871489-103872022 | CpG 40 | q32.33 | intergenic | 2.76E-05 | 0.64 |
| chr14:104174538-104174814 | CpG 30 | q32.33 | intergenic | 5.85E-04 | 0.64 |
| chr14:104310308-104310636 | AKT1 | q32.33 | exon-intron | 3.59E-04 | 0.61 |
| chr14:104756000-104756204 | BRF1 | q32.33 | intron | 5.59E-04 | 0.6 |
| chr14:104922796-104923032 | PACS2 | q32.33 | intron | 6.00E-07 | 0.53 |
| chr14:105036033-105036349 | C14orf80 | q32.33 | exon1 | 4.70E-04 | 0.68 |
| chr14:105392795-105393047 | IGDH | q32.33 | intron | 3.20E-04 | 0.66 |
| chr14:105404537-105404965 | CpG 46 | q32.33 | intergenic | 1.25E-04 | 0.64 |
| chr14:67009927-67010423 | TREM229B | q24.1 | exon1 | 1.18E-04 | 0.66 |
| chr14:98884209-98884849 | CpG 50 | q32.2 | intergenic | 1.10E-04 | 0.65 |
| chr14:99188630-99188941 | HHIPL1 | q32.2 | exon | 3.07E-04 | 0.64 |
| chr15:25922164-25922761 | OCA2 | q13.1 | intron | 1.23E-04 | 0.67 |
| chr15:26051366-26051569 | HERC2 | q13.1 | exon | 2.89E-05 | 0.55 |
| chr15:50874503-50874780 | CpG 24 | q21.2 | intergenic | 6.54E-05 | 0.58 |
| chr15:69294422-69294750 | THSD4 | q23 | exon | 1.23E-04 | 1.82 |
| chr15:73257964-73258258 | CpG 27 | q24.23 | intergenic | 6.20E-05 | 0.57 |
| chr15:73768353-73768655 | CSPG4 | q24.2 | exon | 7.45E-04 | 0.58 |
| chr15:84113157-84114051 | KLHL25 | q25.3 | exon | 6.00E-07 | 0.58 |
| chr15:87225633-87225874 | HAPLN3 | q26.1 | exon | 6.50E-06 | 0.63 |
| chr15:97068317-97068541 | IGF1R | q26.3 | exon | 2.27E-05 | 0.62 |
| chr16:1094941-1095320 | CpG 30 | p13.3 | intergenic | 5.61E-04 | 0.7 |
| chr16:11175393-11175712 | CLEC16A | p13.13 | exon | 3.75E-04 | 0.65 |
| chr16:11277440-11277722 | PRM2 | p13.13 | exon1 | 7.64E-04 | 0.69 |
| chr16:1138522-1138804 | CpG 22 | p13.3 | intergenic | 2.85E-05 | 0.6 |
| chr16:1157129-1157337 | CACNA1H | p13.3 | intron | 5.60E-04 | 0.65 |
| chr16:1389296-1389546 | UNKL | p13.3 | intron-exon-intron | 8.74E-04 | 0.67 |
| chr16:1438350-1438611 | CLCN7 | p13.3 | exon-intron | 7.39E-05 | 0.68 |
| chr16:1447356-1447654 | CLCN7 | p13.3 | intron | 1.00E-06 | 0.5 |
| chr16:1736811-1737230 | MAPK8IP3 | p13.3 | intron-exon | 2.24E-04 | 0.67 |
| chr16:1809078-1809298 | HAGH | p13.3 | intron-exon-intron | 1.12E-04 | 0.6 |
| chr16:1988195-1988755 | ZNF598 | p13.3 | intron-exon-intron | 1.34E-05 | 0.57 |
| chr16:2054026-2054245 | TSC2 | p13.3 | intron | 4.42E-05 | 0.66 |
| chr16:2074260-2074633 | TSC2 | p13.3 | exon | 2.08E-04 | 0.63 |
| chr16:2093240-2093873 | PKD1 | p13.3 | exon | 2.15E-04 | 0.56 |
| chr16:2176713-2176940 | CASKIN1 | p13.3 | exon-intron | 9.81E-04 | 0.61 |
| chr16:2274109-2274348 | ABCA3 | p13.3 | Intron-exon | 8.00E-06 | 0.57 |
| chr16:2279260-2279553 | ABCA3 | p13.3 | Intron-exon | 4.14E-04 | 0.71 |
| chr16:23429074-23429400 | GGA2 | p12.1 | Promoter-exon1 | 2.76E-04 | 1.43 |
| chr16:30501466-30502026 | ZNF785 | p11.2 | exon1 | 2.40E-05 | 0.65 |
| chr16:30701217-30702769 | ZNF 629 | p11.2 | exon1 | 8.48E-05 | 0.65 |
| chr16:3182623-3182913 | CpG 28 | p13.3 | intergenic | 1.02E-03 | 0.71 |
| chr16:3554317-3554602 | NLRC1 | p13.3 | exon | 2.87E-05 | 0.59 |
| chr16:363872-364424 | TREM8A | p13.3 | intron-exon-intron-exon | 1.12E-03 | 0.66 |
| chr16:3664345-3664850 | TRAP1 | p13.3 | exon-intron | 4.89E-05 | 0.62 |
| chr16:3721215-3721419 | CREBBP | p13.3 | Exon | 2.48E-04 | 0.64 |
| chr16:4638220-4638950 | MGRN1 | p13.3 | intron | 5.89E-04 | 0.61 |
| chr16:472468-472670 | RAB11FIP3 | p13.3 | intron-exon | 1.97E-04 | 0.66 |
| chr16:589466-590058 | RAB40C | p13.3 | intron | 9.00E-04 | 0.72 |
| chr16:724368-725035 | NARFL | p13.3 | intron-exon | 3.15E-04 | 0.69 |
| chr16:73820996-73821386 | BCAR1 | q23.1 | exon1 | 1.11E-03 | 0.69 |
| chr16:838973-839174 | CpG 19 | p13.3 | intergenic | 1.03E-04 | 0.6 |
| chr16:84300986-84301579 | C16orf74 | q24.1 | intron-exon-intron | 9.42E-04 | 0.72 |
| chr16:85998888-85999172 | ZCCHC14 | q24.2 | exon1 | 6.88E-04 | 0.65 |
| chr16:86294153-86294408 | LOC100129637 | q24.2 | exon | 5.74E-05 | 0.55 |
| chr16:86300057-86300610 | KLHDC4 | q24.2 | intron-exon | 1.73E-04 | 0.65 |
| chr16:86311509-86311786 | KLHDC4 | q24.2 | intron | 1.99E-04 | 0.63 |
| chr16:86451860-86452067 | SLC7A5 | q24.2 | intron | 1.11E-04 | 0.51 |
| chr16:86601797-86602003 | BANP | q24.2 | intron | 1.18E-04 | 0.58 |
| chr16:86713093-86713609 | CpG 37 | q24.2 | intergenic | 1.84E-04 | 0.57 |
| chr16:87040774-87041015 | ZFPM1 | q24.2 | promoter | 1.48E-04 | 0.63 |
| chr16:87085552-87085830 | ZFPM1 | q24.2 | intron | 1.16E-03 | 0.68 |
| chr16:87087321-87087590 | ZFPM1 | q24.2 | intron | 8.79E-05 | 0.66 |
| chr16:87127111-87129031 | ZFPM1 | q24.2 | exon | 3.57E-04 | 1.66 |
| chr16:87249825-87250199 | MVD | q24.3 | intron-exon | 1.78E-04 | 0.67 |
| chr16:87253084-87253337 | MVD | q24.3 | intron | 6.58E-04 | 0.62 |
| chr16:87309307-87310018 | FAM38A | q24.3 | exon-intron-exon | 5.68E-04 | 0.7 |
| chr16:87435934-87436199 | GALNS | q24.3 | intron | 9.62E-04 | 0.67 |
| chr16:87485761-87486059 | CBFA2T3 | q24.3 | exon-intron | 6.33E-04 | 0.65 |
| chr16:87493703-87494093 | CBFA2T3 | q24.3 | intron | 2.95E-04 | 0.65 |
| chr16:87497865-87498100 | CBFA2T3 | q24.3 | intron | 9.79E-05 | 0.63 |
| chr16:87671345-87672012 | CpG 55 | q24.3 | intergenic | 3.27E-05 | 0.6 |
| chr16:87705282-87705505 | ACSF3 | q24.3 | intron | 9.82E-04 | 0.6 |
| chr16:88015261-88015659 | ANKRD11 | q24.3 | intron | 1.62E-04 | 0.52 |
| chr16:889659-889961 | LMF1 | p13.3 | intron | 1.11E-04 | 0.66 |
| chr16:919423-919664 | LMF1 | p13.3 | intron | 1.16E-04 | 0.6 |
| chr16:919786-920199 | LMF1 | p13.3 | intron | 1.06E-04 | 0.58 |
| chr17:10573514-10574215 | TREM220 | p13.1 | exon | 1.24E-03 | 0.65 |
| chr17:1338105-1338480 | MYO1C | p13.3 | intron | 5.44E-04 | 1.6 |
| chr17:1493492-1494074 | SKARF1 | p13.3 | exon | 7.52E-04 | 1.63 |
| chr17:16379557-16380171 | CpG 49 | p11.2 | intergenic | 5.09E-04 | 0.58 |
| chr17:16428375-16428609 | CpG 22 | p11.2 | intergenic | 3.83E-04 | 0.57 |
| chr17:1786739-1787959 | RTN4RL1 | p13.3 | exon | 6.94E-04 | 0.71 |
| chr17:18815409-18815612 | FAM83G-SLC5A10 | p11.2 | exon | 8.90E-05 | 0.61 |
| chr17:19557675-19558016 | SLC47A2 | p11.2 | exon-intron | 1.18E-03 | 0.66 |
| chr17:205475-206667 | CpG 109 | p13.3 | intergenic | 1.23E-04 | 0.65 |
| chr17:21259243-21260634 | KCNJ12 | p11.2 | exon | 7.20E-06 | 0.6 |
| chr17:22333132-22333426 | CpG 25 | p11.1 | intergenic | 2.73E-04 | 0.61 |
| chr17:24206105-24206445 | ERAL1 | p11.2 | promoter | 6.42E-04 | 0.64 |
| chr17:27847030-27847323 | MYO1D | p11.2 | intron | 2.75E-05 | 0.64 |
| chr17:34610012-34610471 | RPL19 | q12 | promoter-exon1 | 3.92E-04 | 0.64 |
| chr17:35036840-35037445 | PPP1R1B | q12 | promoter | 9.02E-04 | 1.54 |
| chr17:37522926-37523139 | KAT2A | q21.2 | exon | 5.95E-04 | 0.72 |
| chr17:42855779-42856706 | LOC100272146 | q21.32 | exon | 5.79E-04 | 1.75 |
| chr17:51942953-51943498 | CpG 40 | q22 | intergenic | 5.98E-05 | 0.66 |
| chr17:53515753-53516493 | DYNLL2 | q22 | promoter | 1.10E-04 | 1.72 |
| chr17:54093846-54094096 | TEX14 | q22 | intron | 9.25E-04 | 0.65 |
| chr17:55474534-55475210 | CpG 57 | q23.1 | intergenic | 2.53E-05 | 0.6 |
| chr17:59372755-59373051 | SCN4A | q23.3 | exon | 1.08E-03 | 0.68 |
| chr17:659727-660349 | NXN | p13.3 | intron | 1.63E-05 | 0.54 |
| chr17:69879574-69879775 | GPR142 | q25.1 | exon | 1.43E-04 | 0.62 |
| chr17:71979422-71979751 | RHBDF2 | q25.1 | exon | 7.85E-04 | 0.69 |
| chr17:72440305-72440517 | MGAT5B | q25.2 | Exon-intron | 4.39E-04 | 0.64 |
| chr17:72909879-72910122 | 9-Sep | q25.3 | exon | 2.57E-04 | 0.68 |
| chr17:73300921-73301148 | CpG 21 | q25.3 | intergenic | 3.30E-06 | 0.57 |
| chr17:74645522-74645770 | HRNBP3 | q25.3 | intron | 1.58E-04 | 0.59 |
| chr17:75373163-75373460 | CBX2 | q25.3 | exon | 9.72E-04 | 0.68 |
| chr17:75448658-75448963 | CpG 25 | q25.3 | intergenic | 9.02E-04 | 0.68 |
| chr17:75532182-75532480 | TBC1D16 | q25.3 | intron | 1.85E-05 | 0.53 |
| chr17:75537316-75537568 | TBC1D16 | q25.3 | exon-intron | 1.00E-05 | 0.61 |
| chr17:75566440-75566886 | TBC1D16 | q25.3 | intron | 1.15E-03 | 0.66 |
| chr17:75803398-75803605 | SGSH | q25.3 | exon-intron | 8.67E-05 | 0.64 |
| chr17:76413375-76413755 | RPTOR | q25.3 | intron | 1.67E-04 | 0.53 |
| chr17:76478164-76478408 | RPTOR | q25.3 | intron | 4.13E-04 | 0.59 |
| chr17:76554558-76554777 | RPTOR | q25.3 | exon | 2.02E-05 | 0.57 |
| chr17:76587651-76587888 | CHMP6 | q25.3 | exon | 8.11E-04 | 0.65 |
| chr17:77265567-77265920 | HGS | q25.3 | intron | 7.43E-04 | 0.7 |
| chr17:77421925-77423424 | ARHGDIA | q25.3 | promoter | 1.01E-03 | 1.46 |
| chr17:77870407-77871122 | CpG 65 | q25.3 | intergenic | 8.58E-04 | 0.65 |
| chr17:77943479-77943697 | C17orf101 | q25.3 | exon | 1.62E-05 | 0.6 |
| chr17:78134837-78135043 | FOXK2 | q25.3 | intron | 1.38E-05 | 0.55 |
| chr17:78135201-78135408 | FOXK2 | q25.3 | exon-intron | 1.07E-04 | 0.55 |
| chr17:78280668-78281215 | CpG 40 | q25.3 | intergenic | 6.01E-04 | 0.58 |
| chr17:78421259-78421605 | TBCD | q25.3 | intron-exon | 2.84E-04 | 0.61 |
| chr17:78582348-78582681 | B3GNTL1 | q25.3 | intron | 3.67E-05 | 0.56 |
| chr17:78609333-78609584 | CpG 20 | q25.3 | intergenic | 2.03E-04 | 0.6 |
| chr17:78625871-78626248 | CpG 30 | q25.3 | intergenic | 1.95E-04 | 0.61 |
| chr18:10461656-10461867 | APCDD1 | p11.22 | exon | 1.10E-03 | 0.66 |
| chr18:2903059-2903307 | EMILIN2 | p11.31 | exon | 5.00E-07 | 0.57 |
| chr18:53253425-53253706 | CpG 33 | q21.31 | intergenic | 2.27E-04 | 1.56 |
| chr18:70133732-70134724 | CpG 67 | q22.3 | intergenic | 8.06E-04 | 0.7 |
| chr18:72933115-72933448 | MBP | q23 | intron | 7.30E-04 | 0.65 |
| chr18:73987565-73987789 | CpG 18 | q23 | intergenic | 9.64E-04 | 0.64 |
| chr18:74261161-74261432 | CpG 20 | q23 | intergenic | 1.05E-05 | 0.55 |
| chr18:74702742-74702951 | CpG 23 | q23 | intergenic | 1.08E-04 | 0.53 |
| chr18:75214495-75214715 | ATP9B | q23 | intron | 4.55E-04 | 0.69 |
| chr18:75307909-75308534 | NFATC1 | q23 | intron | 4.00E-06 | 0.61 |
| chr18:75309679-75310023 | NFATC1 | q23 | intron-exon | 0.0002313 | 0.66 |
| chr18:75311977-75312201 | NFATC1 | q23 | exon-intron | 1.12E-04 | 0.57 |
| chr18:75341420-75341724 | NFATC1 | q23 | intron | 3.12E-04 | 0.67 |
| chr18:75373572-75374591 | NFATC1 | q23 | intron | 4.20E-06 | 0.63 |
| chr18:75410683-75413214 | Cpg 190 | q23 | intergenic | 2.66E-04 | 0.63 |
| chr18:75580087-75580375 | CTDP1 | q23 | intron | 8.79E-05 | 0.68 |
| chr18:75588333-75588535 | CTDP1 | q23 | intron | 3.33E-05 | 0.57 |
| chr18:75686887-75687844 | CpG 74 | q23 | intergenic | 1.33E-05 | 0.63 |
| chr18:8649443-8649672 | CpG 17 | p11.22 | intergenic | 4.00E-07 | 0.54 |
| chr19:10892933-10893195 | CARM1 | p13.2 | intron-exon-intron | 5.76E-04 | 0.68 |
| chr19:11006589-11006790 | SMARCA4 | p13.2 | exon | 2.03E-05 | 0.57 |
| chr19:11150021-11150248 | KANK2 | p13.2 | exon-intron-exon | 9.86E-04 | 0.67 |
| chr19:1241409-1241637 | EFNA2 | p13.3 | intron | 2.79E-04 | 0.61 |
| chr19:1319814-1320026 | MUM1 | p13.3 | intron | 5.24E-05 | 0.55 |
| chr19:13849204-13849559 | NANOS3 | p13.12 | exon1 | 3.54E-05 | 0.63 |
| chr19:14134429-14135173 | LPNH1 | p13.12 | exon | 3.46E-04 | 0.59 |
| chr19:17472345-17472664 | SLC27A1 | p13.11 | exon-intron-exon | 5.30E-06 | 0.59 |
| chr19:1830910-1831158 | FAM108A1 | p13.3 | exon-intron | 1.28E-05 | 0.62 |
| chr19:1926219-1926570 | CSNK1G2 | p13.3 | intron | 1.17E-03 | 0.69 |
| chr19:1949800-1950332 | BTBD2 | p13.3 | intron | 1.90E-04 | 0.65 |
| chr19:19625631-19625873 | ATP13A1 | p13.11 | exon-intron-exon | 2.76E-04 | 0.64 |
| chr19:21457929-21458716 | CpG 56 | p12 | intergenic | 1.42E-04 | 0.66 |
| chr19:2395300-2395522 | LMNB2 | 13.3 | intron-exon | 4.17E-05 | 0.62 |
| chr19:2601659-2601911 | GNG7 | p13.3 | intron | 4.53E-05 | 0.58 |
| chr19:2653532-2653987 | GNG7 | p13.3 | Promoter | 1.72E-04 | 1.89 |
| chr19:311071-312949 | CpG 183 | p13.3 | intergenic | 8.76E-04 | 0.55 |
| chr19:3483433-3483666 | FZR1 | p13.3 | exon | 2.67E-04 | 0.64 |
| chr19:369898-370191 | SHC2 | p13.3 | exon-intron | 1.16E-04 | 0.69 |
| chr19:40015371-40015792 | LOC400685 | p13.11 | promoter-exon1 | 1.08E-03 | 0.73 |
| chr19:40906468-40906753 | MLL4 | q13.12 | exon | 2.02E-03 | 0.67 |
| chr19:41438552-41438754 | CpG 16 | q13.12 | intergenic | 5.46E-04 | 0.65 |
| chr19:43682103-43682313 | RYR1 | p13.2 | exon | 5.58E-04 | 0.72 |
| chr19:45799137-45799420 | LTBP4 | p13.2 | Exon1 | 4.31E-04 | 1.62 |
| chr19:47276356-47276595 | ZNF 574 | p13.2 | exon | 7.45E-04 | 0.67 |
| chr19:4842191-4842425 | ARRDC5 | p13.3 | exon | 1.68E-04 | 0.67 |
| chr19:4937182-4937405 | KDM4B | p13.3 | intron | 4.58E-04 | 0.57 |
| chr19:5011984-5012242 | KDM4B | p13.3 | intron | 7.32E-04 | 0.68 |
| chr19:5070220-5070445 | KDM4B | p13.3 | intron | 1.64E-05 | 0.6 |
| chr19:5086285-5086545 | KDM4B | p13.3 | intron-exon | 4.61E-05 | 0.61 |
| chr19:52614063-52614589 | MEIS3 | q13.32 | exon1-small part of promoter | 3.60E-04 | 1.7 |
| chr19:53808057-53808453 | FAM83E | p13.33 | Exon1 | 3.57E-04 | 0.66 |
| chr19:53830511-53830897 | DBP | p13.33 | intron-exon | 2.87E-04 | 1.9 |
| chr19:53945500-53946033 | FUT1 | p13.3 | exon | 1.52E-04 | 0.69 |
| chr19:5429299-5429577 | CpG 21 | p13.3 | intergenic | 1.14E-03 | 0.64 |
| chr19:54636220-54636468 | SLC17A7 | p13.33 | intron-exon | 7.15E-04 | 1.69 |
| chr19:55053539-55053793 | PTOV1 | p13.33 | intron-exon-eintron | 6.74E-05 | 0.66 |
| chr19:55518592-55518840 | NCNC3 | p13.33 | exon | 1.87E-04 | 0.67 |
| chr19:5658315-5658524 | LONP1 | p13.3 | intron | 1.12E-03 | 0.64 |
| chr19:57730889-57731732 | ZNF 808 | p13.41 | intron | 1.32E-03 | 0.7 |
| chr19:5782595-5783297 | FUT6 | p13.3 | exon1 | 2.00E-07 | 0.54 |
| chr19:5794864-5795373 | FUT3 | p13.3 | exon | 2.11E-05 | 0.61 |
| chr19:58486223-58486544 | BIRC8 | q13.41 | intron | 1.81E-04 | 0.65 |
| chr19:5990990-5991228 | RFX2 | p13.3 | exon | 9.03E-04 | 0.63 |
| chr19:61304111-61304555 | ZNF 787 | q13.42 | intron | 4.70E-04 | 0.67 |
| chr19:61824970-61825996 | ZNF71 | q13.43 | exon1 | 2.29E-04 | 0.68 |
| chr19:62972738-62973298 | ZNF 586 | q13.42 | exon1+promoter | 1.01E-03 | 0.67 |
| chr19:7594620-7594832 | XAB2 | p13.2 | exon-intron | 9.49E-04 | 0.72 |
| chr19:7904789-7905410 | TIMM44 | p13.2 | exon-intron-exon-intron | 8.76E-04 | 0.73 |
| chr19:8469441-8470225 | PRAM1 | p13.2 | exon | 1.17E-03 | 0.68 |
| chr2:100304211-100305491 | LORNF2 | q11.2 | exon1 | 1.36E-05 | 1.56 |
| chr2:105219925-105220229 | CpG 29 | q12.1 | intergenic | 1.75E-04 | 0.69 |
| chr2:106325547-106325958 | CpG 33 | q12.2 | intergenic | 1.73E-04 | 0.67 |
| chr2:10747175-10747692 | NOL10 | p25.1 | exon -promoter | 8.54E-04 | 0.66 |
| chr2:113524988-113525227 | IL1F8 | q13 | intron | 1.25E-03 | 0.68 |
| chr2:127902567-127902840 | PROC | q14.3 | exon | 5.99E-05 | 0.6 |
| chr2:144863593-144863946 | ZEB2 | q22.3 | exon | 3.83E-04 | 0.64 |
| chr2:176657757-176658041 | CpG 21 | q31.3 | intergenic | 2.47E-04 | 0.65 |
| chr2:1821853-1822144 | MYT1L | p25.3 | intron-exon | 7.75E-04 | 0.66 |
| chr2:196743834-196744628 | STK17B | q32.3 | promoter-exon1 | 4.38E-04 | 1.53 |
| chr2:2168207-2169145 | MYT1L | p25.3 | intron | 2.09E-04 | 0.52 |
| chr2:235616191-235616553 | SH3BP4 | q37.2 | exon | 8.84E-05 | 0.65 |
| chr2:236532391-236532645 | AGAP1 | q37.2 | intron | 7.00E-06 | 0.56 |
| chr2:237945214-237945508 | COL6A3 | q37.3 | exon | 5.98E-04 | 0.67 |
| chr2:239698084-239698390 | HDAC4 | q37.3 | intron-exon-intron | 1.75E-04 | 0.66 |
| chr2:239766440-239766701 | HDAC4 | q37.3 | intron | 4.13E-05 | 0.57 |
| chr2:241052623-241052867 | GPC1 | q37.3 | intron-exon | 1.95E-04 | 0.66 |
| chr2:241229397-241229955 | CpG 59 | q37.3 | intergenic | 2.22E-04 | 0.63 |
| chr2:241827949-241828183 | HDLBP | q37.3 | exon | 1.46E-04 | 0.68 |
| chr2:242678973-242680235 | LOC728323 | q37.3 | promoter-exon1 | 5.70E-06 | 2.08 |
| chr2:2633115-2633365 | CpG 18 | p25.3 | intergenic | 4.70E-06 | 0.56 |
| chr2:37237405-37237906 | EIF2AK2 | p22.2 | promoter | 7.71E-04 | 1.54 |
| chr2:41921441-41921707 | CpG 22 | p21 | intergenic | 1.98E-04 | 0.65 |
| chr2:572818-573385 | CpG 50 | p25.3 | intergenic | 5.67E-04 | 0.64 |
| chr2:60666031-60666258 | CpG 21 | p16.1 | intergenic | 5.91E-05 | 0.63 |
| chr2:6553400-6553611 | CpG 20 | p25.2 | intergenic | 5.52E-04 | 0.63 |
| chr2:81274715-81276043 | CpG 92 | q12 | intergenic | 1.89E-05 | 0.61 |
| chr2:853930-855091 | CpG 150 | p25.3 | intergenic | 4.08E-04 | 0.51 |
| chr2:91624302-91626790 | CpG 182 | p11.1 | intergenic | 4.37E-05 | 0.61 |
| chr2:97717416-97717689 | ZAP70 | p11.2 | exon-intron | 2.02E-04 | 0.63 |
| chr20:13227711-13227947 | ISM1 | p12.1 | exon | 1.34E-04 | 0.64 |
| chr20:23284868-23285201 | CpG 27 | p11.21 | intergenic | 7.04E-05 | 0.62 |
| chr20:30047981-30048439 | XKR1 | p11.21 | exon | 5.22E-04 | 0.65 |
| chr20:30507601-30507802 | C20orf112 | p11.21 | exon | 1.95E-04 | 0.66 |
| chr20:3157045-3157388 | SLC4A11 | p13 | exon-intron-exon | 9.71E-05 | 0.65 |
| chr20:3157813-3158080 | SLC4A11 | p13 | exon-intron-exon | 4.62E-04 | 0.61 |
| chr20:35464951-35465224 | SRC | q11.23 | exon | 2.49E-04 | 0.62 |
| chr20:390530-391317 | TBC1D20 | q13 | promoter-exon1 | 3.33E-04 | 1.61 |
| chr20:46971570-46972079 | ARFGEF2 | p13.13 | promoter-exon1 | 1.65E-04 | 1.62 |
| chr20:50134672-50135139 | ZFP64 | q13.2 | exon | 4.85E-04 | 0.62 |
| chr20:59882170-59882458 | CDH4 | q13.33 | exon-intron | 8.59E-04 | 0.63 |
| chr20:59903475-59903730 | CDH4 | p13.33 | exon-intron | 1.04E-04 | 0.6 |
| chr20:60173841-60174048 | SS18L1 | p13.33 | intron-exon-intron | 1.92E-04 | 0.59 |
| chr20:60403162-60403506 | CABLES2 | q13.33 | exon-intron | 6.60E-06 | 0.57 |
| chr20:60925863-60926192 | COL9A3 | q13.33 | intron-exon | 4.23E-05 | 0.55 |
| chr20:61386379-61386735 | ARFGAP1 | q13.33 | intron-exon | 4.72E-05 | 0.64 |
| chr20:61546504-61546707 | KCNQ2 | q13.33 | exon-intron | 4.19E-04 | 0.64 |
| chr20:61671096-61671397 | PRIC285 | q13.33 | exon | 6.76E-04 | 0.6 |
| chr20:61836693-61836979 | ZGPAT | q13.33 | intron | 7.35E-04 | 0.68 |
| chr20:61891534-61892828 | ZBTB46 | q13.33 | exon-intron | 2.57E-05 | 0.63 |
| chr20:62131405-62131796 | PRPF6 | q13.33 | intron | 1.85E-04 | 0.63 |
| chr21:13990696-13991989 | CpG 86 | q11.2 | intergenic | 5.46E-04 | 0.68 |
| chr21:36181061-36181472 | CpG 42 | q22.12 | intergenic | 2.44E-04 | 0.7 |
| chr21:42049731-42049963 | RIPK4 | q22.3 | exon | 8.34E-05 | 0.64 |
| chr21:42961891-42962098 | PDE9A | q22.3 | intron | 6.68E-04 | 0.62 |
| chr21:43545411-43545640 | CpG 20 | q22.3 | intergenic | 2.61E-04 | 0.65 |
| chr21:43729389-43729687 | CpG 22 | q22.3 | intergenic | 9.80E-06 | 0.59 |
| chr21:44226912-44227270 | AGPAT3 | q22.3 | exon | 9.10E-06 | 0.56 |
| chr21:45234766-45235089 | CpG31 | q22.3 | intergenic | 1.30E-04 | 0.73 |
| chr21:45609558-45609767 | CpG 19 | q22.3 | intergenic | 3.47E-05 | 0.63 |
| chr21:45684794-45685056 | COL18A1 | q22.3 | intron | 1.01E-03 | 0.56 |
| chr21:46111440-46111854 | PCBP3 | q22.3 | intron | 7.22E-04 | 0.65 |
| chr21:46633099-46633549 | PCNT | q22.3 | exon-intron | 2.21E-04 | 0.68 |
| chr21:46637679-46637890 | PCNT | q22.3 | intron | 1.70E-06 | 0.57 |
| chr22:18453363-18453931 | DGCR8-mir1306 | q11.21 | exon1 | 7.71E-04 | 0.69 |
| chr22:20618346-20618560 | PPM1F | q11.22 | intron-exon | 6.05E-04 | 0.68 |
| chr22:21767921-21768431 | GNAZ-RTDR1 | p11.22 | exon | 7.96E-04 | 0.67 |
| chr22:28455352-28455665 | CABP7 | p 12.2 | intron-exon | 1.11E-04 | 0.64 |
| chr22:29886124-29886466 | RNF185 | q12.2 | promoter-exon1 | 2.63E-04 | 0.65 |
| chr22:34150095-34150302 | MCM5 | q12.3 | intron-exon | 8.29E-04 | 0.67 |
| chr22:35908178-35908606 | C1QTNF6 | q13.1 | exon | 3.61E-04 | 0.58 |
| chr22:36575287-36575627 | EIF3L | q13.1 | promoter-exon1 | 1.22E-03 | 0.68 |
| chr22:37481403-37482422 | SUN2 | q13.1 | promoter-exon1 | 1.15E-03 | 1.47 |
| chr22:37569114-37570371 | NPTXR | q13.1 | promoter-exon1 | 3.77E-05 | 1.8 |
| chr22:40071877-40072111 | ZC3H7B | q13.2 | intron-exon | 1.90E-06 | 0.56 |
| chr22:41913446-41913774 | CpG 44 | q13.2 | intergenic | 1.22E-03 | 1.48 |
| chr22:45392952-45393362 | CpG 28 | q13.31 | intergenic | 3.10-04 | 0.66 |
| chr22:45437534-45437778 | GRAMD4 | q13.31 | intron-exon-intron | 1.93E-05 | 0.64 |
| chr22:45448187-45448464 | GRAMD4 | q13.31 | intron-exon-intron | 8.77E-04 | 0.67 |
| chr22:45891866-45892249 | TBC1D22A | q13.31 | intron | 6.99E-04 | 0.69 |
| chr22:47275039-47275255 | FAM19A5 | q13.32 | intron | 1.17E-03 | 0.66 |
| chr22:48084421-48084802 | CpG 34 | q13.32 | intergenic | 3.29E-05 | 0.63 |
| chr22:48269072-48269389 | CpG 30 | q13.33 | intergenic | 8.34E-05 | 0.56 |
| chr22:48581826-48582389 | BRD1 | q13.33 | intron | 3.65E-04 | 0.63 |
| chr22:48603161-48603667 | BRD1 | q13.33 | exon | 1.14E-03 | 0.62 |
| chr22:48607339-48607556 | CpG 27 | q13.33 | intergenic | 2.26E-05 | 2.57 |
| chr22:48806935-48807136 | TTLL8 | q13.33 | intron | 3.45E-04 | 0.64 |
| chr22:48810340-48811480 | TTLL8 | q13.33 | intron-exon | 6.54E-04 | 0.59 |
| chr22:48811993-48812547 | TTLL8 | q13.33 | intron-exon | 2.74E-05 | 0.66 |
| chr22:48950334-48950787 | CpG 46 | q13.33 | intergenic | 1.27E-03 | 1.56 |
| chr22:49001120-49001527 | TUBGCP6 | q13.33 | exon | 2.26E-04 | 0.65 |
| chr22:49068235-49068647 | PLXNB2 | q13.33 | exon-intron-exon | 1.27E-03 | 0.57 |
| chr22:49192024-49192303 | SAPs2 | q13.3 | exon-intron | 3.63E-04 | 0.6 |
| chr22:49246827-49247145 | SBF1 | q13.33 | exon-intron-exon | 9.21E-04 | 0.63 |
| chr22:49249915-49250218 | SBF1 | q13.33 | exon-intron-exon | 3.71E-04 | 0.63 |
| chr3:128190305-128191168 | PLXNA1 | q21.13 | exon | 2.61E-04 | 0.67 |
| chr3:131547261-131547527 | COL29A1 | q22.1 | exon | 3.82E-04 | 0.64 |
| chr3:139211681-139211893 | CLDN18 | q22.3 | promoter-exon1 | 4.00E-06 | 0.59 |
| chr3:15091007-15091223 | ZFYVE20 | p24.3 | exon | 7.95E-04 | 0.65 |
| chr3:159844871-159845169 | GFM1 | q25.32 | promoter-exon1 | 1.28E-03 | 0.6 |
| chr3:187973293-187973504 | CpG 20 | q27.3 | intergenic | 1.85E-05 | 0.63 |
| chr3:188400102-188400447 | RTP1 | q27.3 | exon | 8.14E-05 | 0.65 |
| chr3:188940422-188940639 | BCL6 | q27.3 | intron | 2.76E-04 | 1.98 |
| chr3:197083418-197083786 | TNK2 | q29 | intron-exon-intron | 1.12E-04 | 0.61 |
| chr3:198869395-198869641 | CpG 19 | q29 | intergenic | 5.86E-04 | 0.51 |
| chr3:20056462-20057430 | KAT2B | p24.3 | promoter-exon1 | 1.15E-04 | 1.59 |
| chr3:42218934-42219375 | TRAK1 | p22.1 | exon-intron | 2.00E-07 | 0.51 |
| chr3:42675096-42675359 | ZBTB47 | p22.1 | exon | 1.00E-31 | 0.69 |
| chr3:43380423-43380629 | CpG 18 | p22.1 | intergenic | 1.33E-04 | 0.65 |
| chr3:44493842-44494372 | ZNF445 | p21.32 | promoter-exon1 | 3.93E-05 | 1.55 |
| chr3:50357893-50358314 | ZMYND10 | p21.32 | promoter-exon1 | 2.53E-04 | 0.64 |
| chr3:51405424-51405777 | RBM15B | p21.2 | exon | 1.54E-05 | 0.59 |
| chr3:51724794-51725101 | GRM2 | p21.1 | exon | 2.78E-04 | 0.68 |
| chr3:52714577-52715466 | GLT8D1 | p21.1 | devergent promoter | 3.65E-04 | 1.37 |
| chr3:73515426-73516760 | PDZRN3 | p13 | exon | 5.56E-04 | 0.67 |
| chr4:1212291-1212497 | CTBP1 | p16.3 | intron | 1.26E-04 | 0.51 |
| chr4:1323378-1323676 | MAEA | p16.3 | exon | 2.77E-04 | 0.54 |
| chr4:1356462-1356688 | KIAA1530 | p16.3 | intron | 9.89E-05 | 0.59 |
| chr4:139701184-139701446 | CpG 16 | q31.1 | intergenic | 2.43E-04 | 0.58 |
| chr4:146901960-146902267 | ZNF 827 | q31.22 | intron-exon | 1.12E-04 | 0.65 |
| chr4:1512396-1512673 | CpG 23 | p16.3 | intergenic | 3.35E-04 | 0.67 |
| chr4:154116501-154116810 | FHDC1 | q31.3 | exon | 7.72E-05 | 0.69 |
| chr4:165328833-165329292 | 1-Mar | q32.3 | intron | 1.18E-03 | 0.61 |
| chr4:17225273-17225604 | MED28 | p15.32 | promoter-exon1 | 1.32E-03 | 0.65 |
| chr4:191142227-191143118 | TUBB4Q | q35.2 | exon1-3-part of promoter | 9.16E-05 | 0.65 |
| chr4:2018631-2018942 | CpG 39 | p16.3 | intergenic | 7.03E-04 | 0.63 |
| chr4:25115679-25117174 | CpG 132 | p15.2 | intergenic | 2.37E-04 | 0.66 |
| chr4:2703455-2703818 | FAM 193A | p16.3 | exon | 7.11E-04 | 0.69 |
| chr4:2897851-2898069 | ADD1 | p16.3 | intron | 9.44E-04 | 0.57 |
| chr4:3345629-3345943 | RGS12 | p16.2 | intron | 6.34E-04 | 0.65 |
| chr4:3419448-3419793 | HGFAC | p16.2 | exon-intron-exon | 1.17E-04 | 0.67 |
| chr4:3483246-3483645 | CpG 36 | p.16.2 | intergenic | 2.69E-05 | 0.58 |
| chr4:3489362-3489946 | LRPAP1 | p16.2 | intron-exon-intron | 3.95E-05 | 0.62 |
| chr4:39721906-39722128 | LRPAP1 | p16.2 | exon | 9.00E-07 | 0.57 |
| chr4:519848-520049 | PIGG | p16.3 | intron | 7.74E-05 | 0.61 |
| chr4:56875714-56876904 | KIAA1211 | q12 | exon | 7.41E-04 | 0.7 |
| chr4:62065608-62065843 | LPHN3 | q13.1 | intron | 8.75E-04 | 0.67 |
| chr4:6354434-6355111 | WFS1 | p16.1 | exon | 7.21E-04 | 0.69 |
| chr4:75122817-75123453 | CXCL3 | q13.3 | Promoter-exon1 | 3.54E-04 | 1.7 |
| chr4:77391611-77392084 | FAM47D | q21.1 | promoter | 2.44E-04 | 1.39 |
| chr4:802386-802646 | CPLX1 | p16.3 | intron | 3.40E-04 | 0.66 |
| chr4:860695-860969 | GAK | p16.3 | intron-exon | 4.40E-04 | 0.65 |
| chr5:1140533-1140909 | SLC12A7 | p15.33 | intron | 7.19E-04 | 0.61 |
| chr5:114659885-114660201 | CCDC112 | q22.3 | intron-exon | 4.60E-06 | 1.75 |
| chr5:1330467-1330713 | TERT | p15.33 | intron | 2.89E-04 | 0.69 |
| chr5:169592408-169592807 | C5orf58 | p35.1 | promoter | 1.70E-06 | 0.55 |
| chr5:169863643-169864049 | KCNIP1 | p35.1 | exon | 3.48E-04 | 1.47 |
| chr5:176696721-176697148 | LMAN | p35.3 | exon-intron-exon | 7.37E-04 | 0.64 |
| chr5:176793468-176793704 | GRK6 | p35.3 | intron-exon | 6.76E-04 | 0.66 |
| chr5:177949164-177950276 | COL23A1 | q35.3 | Promoter-exon1 | 5.11E-04 | 1.51 |
| chr5:2233251-2233467 | CpG 18 | p15.33 | intergenic | 1.10E-03 | 0.72 |
| chr5:234725-234944 | PLEKHG4B | p15.33 | exon-intron | 3.60E-04 | 0.66 |
| chr5:26705305-26705661 | CpG 28 | p14.1 | intergenic | 9.15E-05 | 0.68 |
| chr5:28845157-28846277 | CpG 76 | p14.1 | intergenic | 1.06E-03 | 0.67 |
| chr5:360286-360592 | AHRR-PDCD6 | p15.33 | intron | 1.79E-03 | 0.59 |
| chr5:581383-581982 | CpG 69 | p15.33 | intergenic | 1.20E-06 | 1.92 |
| chr5:730813-731135 | TPPP | p15.33 | intron-exon | 2.71E-04 | 0.61 |
| chr6:109718142-109718923 | CpG 56 | q21 | intergenic | 8.54E-04 | 0.68 |
| chr6:139055256-139056574 | CpG 122 | q23.3 | intergenic | 1.13E-04 | 1.46 |
| chr6:139529852-139530067 | HECA | q24.1 | exon | 2.25E-04 | 0.69 |
| chr6:15625261-15625536 | JARID2 | p22.3 | intron-exon | 1.30E-03 | 0.63 |
| chr6:160431926-160432455 | IGF2R | q25.3 | intron | 2.85E-05 | 0.51 |
| chr6:166059991-166060283 | CpG 22 | q27 | intergenic | 1.43E-03 | 0.61 |
| chr6:168095365-168095577 | MLLT4 | q27 | exon | 1.23E-04 | 0.63 |
| chr6:168272831-168273156 | CpG 23 | q27 | intergenic | 2.98E-04 | 0.6 |
| chr6:168376555-168376767 | CpG 18 | q27 | intergenic | 1.73E-04 | 0.6 |
| chr6:169383728-169383945 | THBS2 | q27 | inron-exon | 1.24E-04 | 0.63 |
| chr6:169390471-169390972 | THBS2 | q27 | exon | 2.45E-05 | 0.61 |
| chr6:170267707-170268038 | cPg 25 | q27 | intergenic | 1.32E-05 | 0.58 |
| chr6:170295849-170296860 | CpG 69 | q27 | intergenic | 6.31E-05 | 0.59 |
| chr6:170432516-170432834 | CpG 29 | q27 | intergenic | 8.97E-05 | 0.59 |
| chr6:25990306-25990539 | CpG 24 | p22.2 | intergenic | 1.04E-04 | 0.66 |
| chr6:26232472-26232761 | HIST1H2AC | p22.1 | exon | 5.00E-05 | 0.65 |
| chr6:26354794-26355079 | HIST1H4G | p22.1 | Promoter-exon1 | 8.71E-04 | 0.72 |
| chr6:27222201-27223160 | HIST1H2BK-HIST1H2AH | p22.1 | promoter | 6.12E-04 | 0.65 |
| chr6:33504028-33504274 | SYNGAP1 | p21.32 | intron | 5.50E-06 | 2.12 |
| chr6:34607291-34607558 | PACSIN1 | p21.31 | intron-exon | 1.22E-03 | 0.65 |
| chr6:35862691-35862892 | C6orf127 | p21.31 | intron-exon | 8.76E-04 | 0.71 |
| chr6:35996457-35997038 | SRPK1 | p21.31 | promoter-exon1 | 8.45E-04 | 1.4 |
| chr6:37037815-37038113 | PI16 | p21.2 | intron | 2.52E-04 | 0.66 |
| chr6:38715755-38716126 | BTBD9 | p21.2 | promoter-exon1 | 6.01E-04 | 1.48 |
| chr6:7931332-7931846 | PIP5K1P1 | p24.3 | exon | 1.26E-04 | 0.66 |
| chr6:832605-832908 | CpG 23 | p25.3 | intergenic | 4.47E-04 | 0.63 |
| chr7:105539339-105540384 | SYPL1 | q22.2 | promoter-exon1 | 7.45E-04 | 1.53 |
| chr7:134568859-134569194 | STRA8 | q33 | intron | 1.94E-05 | 0.62 |
| chr7:148825072-148826208 | ZNF 746 | q36.1 | promoter-exon1 | 1.19E-03 | 1.91 |
| chr7:1490067-1490502 | INTS1 | p22.3 | intron-exon-intron | 8.43E-04 | 0.66 |
| chr7:1542262-1542519 | MAFK | p22.3 | intron | 7.69E-05 | 0.66 |
| chr7:155266363-155266587 | RBM33 | q36.3 | exon | 5.79E-04 | 0.66 |
| chr7:155548791-155549110 | CpG 22 | q36.3 | intergenic | 6.20E-04 | 0.64 |
| chr7:156525439-156525722 | CpG 22 | q36.3 | intergenic | 9.39E-04 | 0.69 |
| chr7:156899695-156900109 | DNAJB6 | q36.3 | intron | 1.47E-04 | 0.6 |
| chr7:156901555-156901769 | DNAJB6 | q36.3 | exon | 1.36E-04 | 0.63 |
| chr7:156909359-156909710 | CpG 23 | q36.3 | intergenic | 3.85E-04 | 0.69 |
| chr7:157106662-157106907 | PTPRN2 | q36.3 | intron-exon | 6.69E-04 | 0.59 |
| chr7:157146535-157146969 | PTPRN2 | q36.3 | intron | 5.97E-04 | 0.65 |
| chr7:157387786-157388119 | PTPRN2 | q36.3 | intron | 1.02E-03 | 0.53 |
| chr7:157942889-157943258 | PTPRN2 | q36.3 | intron | 1.14E-03 | 0.62 |
| chr7:157974134-157974495 | PTPRN2 | q36.3 | intron | 2.92E-05 | 0.61 |
| chr7:158362561-158362766 | WDR60 | q36.3 | intron | 1.06E-03 | 0.55 |
| chr7:158579128-158579356 | VIPR2 | q36.3 | intron | 2.00E-05 | 0.63 |
| chr7:16427303-16427790 | ISPD | p21.1 | promoter-exon1 | 0.000483 | 1.55 |
| chr7:16532777-16533199 | CpG 34 | p21.1 | intergenic | 0.0003347 | 0.75 |
| chr7:1832069-1832294 | MAD1L1 | p21.1 | intron | 2.14E-04 | 0.59 |
| chr7:1857356-1857614 | MAD1L1 | p21.1 | intron | 5.20E-06 | 0.55 |
| chr7:1869288-1869585 | MAD1L1 | p21.1 | intron | 6.14E-04 | 0.68 |
| chr7:1916805-1917008 | MAD1L1 | q22.3 | intron | 1.27E-05 | 0.57 |
| chr7:1958440-1958648 | MAD1L1 | p21.1 | intron | 6.18E-04 | 0.62 |
| chr7:2011109-2011314 | MAD1L1 | p21.1 | intron | 6.38E-05 | 0.65 |
| chr7:2152297-2152731 | MAD1L1 | p21.1 | intron | 1.02E-03 | 0.63 |
| chr7:2259802-2260102 | CpG 23 | p22.2 | intergenic | 5.88E-05 | 0.64 |
| chr7:2386334-2386866 | EIF3B | p22.2 | exon1 | 4.97E-04 | 0.69 |
| chr7:2532398-2532640 | LFNG | p22.2 | exon-intron | 5.90E-06 | 0.58 |
| chr7:254856-255122 | CpG 29 | p22.3 | intergenic | 1.22E-04 | 0.67 |
| chr7:301534-302448 | FAM20C | p22.3 | intron | 5.35E-04 | 0.6 |
| chr7:4314233-4314543 | CpG 25 | p22.2 | intergenic | 9.24E-04 | 0.72 |
| chr7:4822516-4822738 | RADIL | p22.1 | exon-intron | 1.02E-03 | 0.72 |
| chr7:4823384-4823692 | RADIL | p22.1 | exon-intron | 7.12E-04 | 0.67 |
| chr7:4889233-4890102 | RADIL | q22.1 | Promoter-exon1 | 1.94E-04 | 1.57 |
| chr7:50503234-50503447 | DDC | p12.2 | intron | 4.55E-04 | 0.68 |
| chr7:5234274-5234636 | WIP12 | p22.1 | exon-intron | 6.67E-05 | 0.69 |
| chr7:5357849-5358098 | DDC | p12.2 | exon.intron | 4.32E-04 | 0.53 |
| chr7:5487894-5488141 | FBXL18 | p22.1 | exon | 5.00E-07 | 0.57 |
| chr7:5561179-5561604 | CpG 43 | q22.1 | intergenic | 6.00E-04 | 1.49 |
| chr7:6176455-6177243 | CYTH3 | p22.1 | intron-exon-intron-exon | 1.32E-03 | 0.64 |
| chr7:909233-909466 | ADAP1 | p22.3 | intron | 1.81E-04 | 0.64 |
| chr8:1120168-1120390 | CpG 18 | p23.3 | intergenic | 1.03E-04 | 0.61 |
| chr8:11644725-11645178 | GATA4 | p23.1 | intron-exon | 6.66E-05 | 0.66 |
| chr8:1233078-1233871 | CpG 58 | p23.3 | intergenic | 7.29E-05 | 0.53 |
| chr8:1250830-1253745 | CpG 192 | p23.3 | intergenic | 4.80E-05 | 0.51 |
| chr8:1385342-1385928 | CpG 50 | p23.3 | intergenic | 2.38E-04 | 0.68 |
| chr8:141628346-141628550 | EIF2C2 | q24.3 | intron-exon | 2.90E-04 | 0.69 |
| chr8:142300845-142301156 | SLC45A4 | q24.3 | exon-intron | 2.14E-05 | 0.56 |
| chr8:142506215-142506454 | PTP4A3 | q24.3 | exon-intron | 3.69E-04 | 0.64 |
| chr8:143376868-143377185 | TSNARE1 | q24.3 | intron | 7.34E-05 | 0.6 |
| chr8:145183285-145183552 | OPLAH | q24.3 | exon-intron-exon | 1.13E-04 | 0.69 |
| chr8:145632951-145633191 | NFKBIL2 | q24.3 | exon-intron | 4.85E-04 | 0.71 |
| chr8:145784727-145785004 | ARHGAP39 | q24.3 | intron | 8.86E-04 | 0.71 |
| chr8:145794298-145794504 | ARHGAP39 | q24.3 | intron | 1.19E-03 | 0.57 |
| chr8:1767027-1767228 | ARHGEF10 | p23.3 | intron | 1.06E-03 | 0.6 |
| chr8:1818155-1818415 | ARHGEF10 | p23.3 | intron-exon-intron | 4.82E-04 | 0.64 |
| chr8:1908966-1910279 | KBTBD11 | p23.3 | promoter-exon1 | 3.49E-04 | 1.57 |
| chr8:2034098-2034370 | MYOM2 | p23.3 | exon-intron | 3.74E-04 | 0.66 |
| chr8:21652358-21652615 | GFRA2 | p21.3 | exon | 1.50E-04 | 0.61 |
| chr8:28629516-28629759 | EXTL3 | p21.1 | exon | 1.09E-05 | 0.54 |
| chr8:33699906-33700142 | CpG 18 | p12 | intergenic | 1.11E-04 | 0.55 |
| chr8:779923-780798 | CpG 87 | p23.3 | intergenic | 4.66E-04 | 0.56 |
| chr8:949722-950154 | CpG 33 | p23.3 | intergenic | 6.89E-05 | 0.61 |
| chr8:99906637-99907112 | STK3 | q22.2 | exon1-part of promoter | 3.76E-04 | 1.72 |
| chr9:100787697-100787904 | COL15A1 | q22.33 | exon | 2.96E-04 | 0.67 |
| chr9:111123154-111123370 | CpG20 | q31.3 | intergenic | 2.74E-04 | 1.68 |
| chr9:115900294-115900516 | KIF12 | q32 | intron | 9.33E-05 | 1.53 |
| chr9:125172819-125173074 | CRB2 | q33.2 | exon | 6.43E-04 | 0.66 |
| chr9:126945496-126945768 | SCAI | q33.2 | promoter-exon1 | 3.13E-04 | 1.67 |
| chr9:128037951-128038247 | Cpg27 | q33.2 | intergenic | 4.41E-04 | 0.63 |
| chr9:129723790-129724191 | PIP5KL1 | q34.11 | exon | 1.27E-03 | 0.62 |
| chr9:131670145-131670644 | USP20 | q34.11 | exon-intron | 9.69E-04 | 0.68 |
| chr9:13313112-13313350 | CpG 19 | p23 | intergenic | 4.06E-04 | 0.68 |
| chr9:135090620-135090934 | CpG 23 | q34.2 | intergenic | 2.68E-04 | 0.59 |
| chr9:136009229-136009476 | WDR5 | q34.2 | intron-exon | 5.44E-05 | 0.61 |
| chr9:137207911-137208115 | CpG 16 | q34.2 | intergenic | 4.09E-04 | 0.64 |
| chr9:138106469-138107288 | NACC2 | q34.3 | intron | 3.24E-04 | 0.68 |
| chr9:138236486-138236814 | LHX3 | q34.2 | promoter | 9.64E-05 | 1.51 |
| chr9:138513010-138513681 | NOTCH1 | q34.3 | intron-exon-intron-exon | 1.40E-05 | 0.6 |
| chr9:138846038-138846261 | C9orf86 | q34.3 | exon-intron | 1.80E-06 | 0.6 |
| chr9:139832752-139833946 | EHMT1 | q34.3 | intron | 2.72E-04 | 0.6 |
| chr9:21549133-21549816 | LOC554202 | p21.3 | promoter-exon1 | 9.91E-04 | 1.57 |
| chr9:69642237-69643493 | CpG 152 | q12 | intergenic | 1.30E-04 | 0.56 |
| chr9:89629425-89629912 | CpG 40 | q22.1 | intergenic | 8.27E-05 | 0.68 |
| chrX:100332896-100333393 | CpG 44 | p22.1 | intergenic | 2.30E-04 | 1.67 |
| chrX:152191547-152192361 | CpG 57 | q28 | intergenic | 1.76E-04 | 0.56 |
| chrX:153347546-153347968 | PLXNA3 | q28 | exon-intron-exon | 1.15E-03 | 0.7 |
| chrX:2845949-2846236 | ARSD | p22.33 | exon | 3.15E-04 | 0.59 |
| chrY:251505-252418 | PPP2R3B | p11.32 | intron | 2.38E-04 | 0.66 |
| chrY:2537106-2537697 | NCRNA00103 | p11.31 | promoter | 2.22E-04 | 1.68 |
| chrY:287371-289648 | CpG209 | p11.32 | intergenic | 5.40E-04 | 0.59 |
| **hypermethylated CpG islands** | | | | | |
| chr11:1214160-1214382 | MUC5B | p15.5 | exon | 5.00E-07 | 0.47 |
| chr11:579803-580121 | PHRF1 | p15.5 | intron | 9.02E-05 | 0.44 |
| chr12:34382273-34382538 | CpG 22 | p11.1 | intergenic | 6.28E-05 | 0.47 |
| chr13:112169630-112169865 | CpG 17 | q34 | intergenic | 6.00E-07 | 0.45 |
| chr16:86589657-86592469 | BANP | q24.2 | intron | 3.83E-04 | 0.5 |
| chr18:74365675-74366095 | CpG 27 | q23 | intergenic | 5.20E-06 | 0.46 |
| chr2:134665537-134666197 | CpG 57 | q21.2 | intergenic | 3.00E-07 | 0.5 |
| chr2:3268433-3268747 | TSSC1 | p25.3 | intron | 1.00E-07 | 0.47 |
| chr21:43350723-43351134 | CBS | q22.3 | intron | 1.05E-05 | 0.48 |
| chr21:44031007-44031479 | CpG 35 | q22.3 | intergenic | 8.00E-07 | 0.48 |
| chr21:45990214-45991354 | CpG 115 | q22.3 | intergenic | 8.40E-06 | 0.46 |
| chr21:9906603-9906958 | CpG 46 | p11.2 | intergenic | 8.61E-04 | 0.44 |
| chr22:47884166-47884381 | CpG 16 | q13.32 | intergenic | 3.34E-05 | 0.47 |
| chr4:888030-888532 | GAK | p16.3 | intron-exon | 8.00E-07 | 0.47 |
| chr6:170371513-170372597 | CpG 68 | q27 | intergenic | 1.56E-04 | 0.46 |
| chr7:157132785-157135482 | PTPRN2 | q36.3 | intron | 2.06E-05 | 0.5 |
| chr7:157270446-157270703 | PTPRN2 | q36.3 | intron | 7.00E-07 | 0.46 |
| chr8:1337546-1337984 | CpG 28 | p23.3 | intergenic | 7.15E-05 | 0.38 |
| chr9:137296531-137296804 | CpG 24 | q34.3 | intergenic | 1.37E-05 | 0.5 |
| chrY:106504-107721 | CpG 96 | p11.32 | intergenic | 2.78E-05 | 0.37 |
