# Supplementary material for: Global Methylation Patterns in Idiopathic Pulmonary Fibrosis
Source: PLoS One. 2012 Apr 10;7(4):e33770. doi: 10.1371/journal.pone.0033770 (PMC3323629; doi:10.1371/journal.pone.0033770)
Supplement: Table S3 — Functional Analysis of the 55 Differentialy methylated promoters in IPF. (DOC) [file pone.0033770.s003.doc]

**Table S3. Functional Analysis of the 55 Differentialy methylated promoters in IPF.**

| **functional category** | **count** | **p-value range** | **Genes**  (genes in bold overlap between IPF and Cancer) |
| --- | --- | --- | --- |
| Cellular Assembly and Organization | 6 | 2,36E-03 - 4,84E-02 | GGA2, **KAT2B**, GFM1, ARFGEF2, ARHGDIA, **EIF2AK2** |
| Cellular Growth and Proliferation | 5 | 4,71E-03 - 4,62E-02 | CXCL3, DIRAS3, **MSI1**, STK3, GNG7 |
| Cell Morphology | 5 | 7,06E-03 - 4,84E-02 | GGA2, BMP8B, ARHGDIA, **EIF2AK2**, STK17B |
| Cancer | 15 | 9,41E-03 - 3,27E-02 | C10orf54**, SRPK1**, DIRAS3, ARFGEF2, STK3, **KAT2B**, CXCL3, TUBB4Q, COL23A1, **MSI1**, **RPL19**, ERAL1, ARHGDIA, **EIF2AK2**, STK17B |
| Cell Signaling | 5 | 1,03E-02 - 4,84E-02 | CXCL3, **KAT2B**, ARHGDIA, SCAI, GNG7 |
| Gene Expression | 4 | 1,41E-02 - 4E-02 | CXCL3, **KAT2B**, DIRAS3, **EIF2AK2** |
| Cell Death | 5 | 1,92E-02 - 4,62E-02 | CXCL3, **SRPK1**, PPP1R1B, DIRAS3, ARHGDIA |
