# Supplementary material for: Global Methylation Patterns in Idiopathic Pulmonary Fibrosis
Source: PLoS One. 2012 Apr 10;7(4):e33770. doi: 10.1371/journal.pone.0033770 (PMC3323629; doi:10.1371/journal.pone.0033770)
Supplement: Table S4 — Differentially methylated CpG islands overlapping between IPF and cancer. (DOC) [file pone.0033770.s004.doc]

**Table S4. Differentially methylated CpG islands overlapping between IPF and cancer.**

| **CpG island location** | **Gene Symbol** | **locus** | **DNA region** |
| --- | --- | --- | --- |
| chr1:1016240-1016488 | C1orf159 | p36.33 | intron |
| chr1:10630397-10630727 | CASZ1 | p36.22 | exon1 |
| chr1:1151822-1152144 | SDF4 | p36.33 | intron |
| chr1:1445165-1445755 | ATAD3A | p36.33 | exon-intron |
| chr1:145846762-145847106 | GJA8 | q21.1 | exon1 |
| chr1:146184780-146185539 | NBPF11 | q21.1 | intron |
| chr1:151928827-151929063 | NPR1 | q21.3 | exon-intron |
| chr1:154808723-154809126 | IQGAP3 | q23.1 | promoter-exon1-intron |
| chr1:15926203-15926562 | PLEKHM2 | p36.21 | exon |
| chr1:15930884-15931092 | PLEKHM2 | p36.21 | exon-intron |
| chr1:16347698-16347911 | EPHA2 | p36.13 | exon |
| chr1:16924078-16924478 | CpG 38 | p36.13 | intergenic |
| chr1:19053532-19054056 | TAS1R2 | p36.13 | exon |
| chr1:19082213-19082501 | ALDH4A1 | p36.13 | exon-intron-exon |
| chr1:2056227-2056526 | PRKCZ | p36.33 | intron |
| chr1:208923682-208923885 | KCNH1 | q32.2 | exon |
| chr1:22328610-22328812 | WNT4 | p36.12 | exon-intron |
| chr1:224141773-224142303 | LEFTY1 | q42.12 | exon-intron-exon |
| chr1:226528508-226528762 | OBSCN | q42.13 | exon |
| chr1:226540458-226540666 | OBSCN | q42.13 | exon |
| chr1:226614700-226614953 | OBSCN | q42.13 | exon-intron |
| chr1:2294019-2294236 | MORN1 | p36.33 | intron |
| chr1:242080425-242081215 | CpG 70 | q44 | intergenic |
| chr1:2429986-2430338 | PANK4 | p36.32 | exon |
| chr1:243915162-243915726 | KIF26B | q44 | intron-exon |
| chr1:2441077-2441411 | PANK4 | p36.32 | exon-intron |
| chr1:28157607-28157891 | SMPDL3B | p35.3 | exon |
| chr1:2908225-2908613 | CpG 34 | p36.32 | intergenic |
| chr1:33863192-33863441 | CSMD2 | p35.1 | exon |
| chr1:3501569-3501978 | MEGF6 | p36.32 | intron-exon-intron |
| chr1:43125372-43125581 | CpG 15 | p34.2 | intergenic |
| chr1:43126534-43126757 | CpG 17 | p34.2 | intergenic |
| chr1:5859744-5859979 | NPHP4 | p36.31 | exon |
| chr1:76312735-76313241 | ST6GALNAC3 | p31.1 | promoter-exon1 |
| chr1:7645987-7647762 | GAMTA1 | p36.23 | exon1 |
| chr1:8308482-8308755 | SLC45A1 | p36.23 | exon-intron |
| chr1:8685592-8686136 | RERE | p36.23 | intron |
| chr1:9247274-9247603 | H6PD | p36.22 | exon1 |
| chr10:126676541-126676882 | CTBP2 | q26.13 | exon-intron |
| chr10:133601389-133601624 | PPP2R2D | q26.3 | intron |
| chr10:133866033-133866334 | DPYSL4 | q26.3 | intron-exon |
| chr10:134209523-134209770 | INPP5A) | q26.3 | intron |
| chr10:134474044-134474265 | C10orf92 | q26.3 | intron |
| chr10:134522359-134522594 | CpG16 | q26.3 | downstream NKX6-2 |
| chr10:134870608-134870922 | KNDC1 | q26.3 | exon-intron |
| chr10:13556171-13556753 | BEND7 | p13 | intron |
| chr10:15294604-15294858 | FAM171A1 | p13 | exon1 |
| chr10:38009142-38010356 | CpG 82 | p11.21 | intergenic |
| chr10:70678278-70678484 | HKDC1 | q21.3 | exon |
| chr10:73203097-73203498 | CDH23 | q22.1 | intronCDH23, promoter C10orf54 |
| chr10:99319950-99320194 | UBTD1 | q24.1 | exon |
| chr11:1214160-1214382 | MUC5B | p15.5 | exon |
| chr11:2774563-2774957 | KCNQ1 | p15.5 | intron |
| chr11:462781-463065 | PTDSS2 | p15.5 | intron |
| chr11:579803-580121 | PHRF1 | p15.5 | intron |
| chr11:601691-601943 | PHRF1 | p15.5 | exon1 |
| chr11:64928945-64929172 | FRMD8 | q13.11 | exon |
| chr11:67189331-67189627 | ALDH3B2 | q13.2 | exon |
| chr11:67935494-67935738 | LRP5 | q13.2 | exon |
| chr11:67949086-67949374 | LRP5 | q13.2 | exon |
| chr11:69177991-69178333 | CCND1 | q13.2 | exon |
| chr11:69334289-69334654 | FGF3 | q13.3 | exon |
| chr12:119018935-119019400 | RAB35 | q24.23 | exon1 |
| chr12:119290634-119291409 | MSI1 | q24.31 | promoter-exon1-intron |
| chr12:120739290-120739877 | SETD1B | q24.31 | exon-intron |
| chr12:123354512-123354837 | FAM101A | q24.31 | intron |
| chr12:123789473-123789703 | CpG 18 | q24.31 | intergenic |
| chr12:130859396-130859769 | CpG 36 | q24.33 | intergenic |
| chr12:131355831-131356429 | GALNT9 | q24.33 | intron |
| chr12:131562753-131562976 | CpG 21 | q24.33 | intergenic |
| chr12:131650377-131650707 | FBRSL1 | q24.33 | intron |
| chr12:34382273-34382538 | CpG 22 | p11.1 | intergenic |
| chr12:6257915-6258429 | CpG 64 | p13.31 | intergenic |
| chr13:109962285-109962548 | COL4A2 | q34 | exon |
| chr13:112522081-112522353 | ATP11A | q34 | intron-exon-intron |
| chr13:113133424-113133683 | ADPRHL1 | q34 | intron |
| chr13:113246319-113246553 | TMCO3 | q34 | intron |
| chr13:113576152-113576502 | GAS6 | q34 | intron-exon-intron |
| chr13:113804437-113804639 | RASA3 | q34 | intron |
| chr13:113897143-113897632 | RASA3 | q34 | intron |
| chr13:31318630-31319274 | EEF1DP3 | p13.1 | promoter |
| chr14:104174538-104174814 | CpG 30 | q32.33 | intergenic |
| chr14:104310308-104310636 | AKT1 | q32.33 | exon-intron |
| chr14:104756000-104756204 | BRF1 | q32.33 | intron |
| chr14:104922796-104923032 | PACS2 | q32.33 | intron |
| chr14:105036033-105036349 | C14orf80 | q32.33 | exon1 |
| chr14:105392795-105393047 | IGDH | q32.33 | intron |
| chr14:67009927-67010423 | TREM229B | q24.1 | exon1 |
| chr14:99188630-99188941 | HHIPL1 | q32.2 | exon |
| chr15:73768353-73768655 | CSPG4 | q24.2 | exon |
| chr15:84113157-84114051 | KLHL25 | q25.3 | exon |
| chr15:87225633-87225874 | HAPLN3 | q26.1 | exon |
| chr16:1094941-1095320 | CpG 30 | p13.3 | intergenic |
| chr16:11175393-11175712 | CLEC16A | p13.13 | exon |
| chr16:11277440-11277722 | PRM2 | p13.13 | exon1 |
| chr16:1138522-1138804 | CpG 22 | p13.3 | intergenic |
| chr16:1157129-1157337 | CACNA1H | p13.3 | intron |
| chr16:1389296-1389546 | UNKL | p13.3 | intron-exon-intron |
| chr16:1438350-1438611 | CLCN7 | p13.3 | exon-intron |
| chr16:1447356-1447654 | CLCN7 | p13.3 | intron |
| chr16:1736811-1737230 | MAPK8IP3 | p13.3 | intron-exon |
| chr16:1809078-1809298 | HAGH | p13.3 | intron-exon-intron |
| chr16:1988195-1988755 | ZNF598 | p13.3 | intron-exon-intron |
| chr16:2054026-2054245 | TSC2 | p13.3 | intron |
| chr16:2074260-2074633 | TSC2 | p13.3 | exon |
| chr16:2093240-2093873 | PKD1 | p13.3 | exon |
| chr16:2176713-2176940 | CASKIN1 | p13.3 | exon-intron |
| chr16:2274109-2274348 | ABCA3 | p13.3 | intron, part exon |
| chr16:2279260-2279553 | ABCA3 | p13.3 | intron, part exon |
| chr16:30501466-30502026 | ZNF785 | p11.2 | Exon1 |
| chr16:30701217-30702769 | ZNF 629 | p11.2 | exon1 |
| chr16:3182623-3182913 | CpG 28 | p13.3 | intergenic |
| chr16:3554317-3554602 | NLRC1 | p13.3 | exon |
| chr16:363872-364424 | TREM8A | p13.3 | intron-exon-intron-exon |
| chr16:3664345-3664850 | TRAP1 | p13.3 | exon-intron |
| chr16:3721215-3721419 | CREBBP | p13.3 | exon |
| chr16:4638220-4638950 | MGRN1 | p13.3 | intron |
| chr16:472468-472670 | RAB11FIP3 | p13.3 | intron-exon |
| chr16:589466-590058 | RAB40C | p13.3 | intron |
| chr16:724368-725035 | NARFL | p13.3 | intron-exon |
| chr16:73820996-73821386 | BCAR1 | q23.1 | exon1 |
| chr16:84300986-84301579 | C16orf74 | q24.1 | intron-exon-intron |
| chr16:86294153-86294408 | LOC100129637 | q24.2 | exon |
| chr16:86300057-86300610 | KLHDC4 | q24.2 | intron-exon |
| chr16:86311509-86311786 | KLHDC4 | q24.2 | intron |
| chr16:86713093-86713609 | CpG 37 | q24.2 | intergenic |
| chr16:87040774-87041015 | ZFPM1 | q24.2 | promoter |
| chr16:87087321-87087590 | ZFPM1 | q24.2 | intron |
| chr16:87249825-87250199 | MVD | q24.3 | intron-exon |
| chr16:87253084-87253337 | MVD | q24.3 | intron |
| chr16:87309307-87310018 | FAM38A | q24.3 | exon-intron-exon |
| chr16:87493703-87494093 | CBFA2T3 | q24.3 | intron |
| chr16:87671345-87672012 | CpG 55 | q24.3 | intergenic |
| chr16:889659-889961 | LMF1 | p13.3 | intron |
| chr16:919423-919664 | LMF1 | p13.3 | intron |
| chr16:919786-920199 | LMF1 | p13.3 | intron |
| chr17:10573514-10574215 | TREM220 | p13.1 | exon |
| chr17:1338105-1338480 | MYO1C | p13.3 | intron |
| chr17:1786739-1787959 | RTN4RL1 | p13.3 | exon |
| chr17:205475-206667 | CpG 109 | p13.3 | intergenic |
| chr17:21259243-21260634 | KCNJ12 | p11.2 | exon |
| chr17:27847030-27847323 | MYO1D | p11.2 | intron |
| chr17:34610012-34610471 | RPL19 | q12 | exon1-part of promoter |
| chr17:51942953-51943498 | CpG 40 | q22 | intergenic |
| chr17:54093846-54094096 | TEX14 | q22 | intron |
| chr17:55474534-55475210 | CpG 57 | q23.1 | intergenic |
| chr17:59372755-59373051 | SCN4A | q23.3 | exon |
| chr17:659727-660349 | NXN | p13.3 | intron |
| chr17:69879574-69879775 | GPR142 | q25.1 | exon |
| chr17:72909879-72910122 | 9-Sep | q25.3 | exon |
| chr17:74645522-74645770 | HRNBP3 | q25.3 | intron |
| chr17:75373163-75373460 | CBX2 | q25.3 | exon |
| chr17:75448658-75448963 | CpG 25 | q25.3 | intergenic |
| chr17:75537316-75537568 | TBC1D16 | q25.3 | exon-intron |
| chr17:75566440-75566886 | TBC1D16 | q25.3 | intron |
| chr17:76413375-76413755 | RPTOR | q25.3 | intron |
| chr17:76478164-76478408 | RPTOR | q25.3 | intron |
| chr17:76554558-76554777 | RPTOR | q25.3 | exon |
| chr17:76587651-76587888 | CHMP6 | q25.3 | exon |
| chr17:77265567-77265920 | HGS | q25.3 | intron |
| chr17:77870407-77871122 | CpG 65 | q25.3 | intergenic |
| chr17:77943479-77943697 | C17orf101 | q25.3 | exon |
| chr17:78134837-78135043 | FOXK2 | q25.3 | intron |
| chr17:78135201-78135408 | FOXK2 | q25.3 | exon-intron |
| chr17:78280668-78281215 | CpG 40 | q25.3 | intergenic |
| chr17:78421259-78421605 | TBCD | q25.3 | intron-exon |
| chr17:78582348-78582681 | B3GNTL1 | q25.3 | intron |
| chr17:78609333-78609584 | CpG 20 | q25.3 | intergenic |
| chr17:78625871-78626248 | CpG 30 | q25.3 | intergenic |
| chr18:53253425-53253706 | CpG 33 | q21.31 | intergenic |
| chr18:70133732-70134724 | CpG 67 | q22.3 | intergenic |
| chr18:72933115-72933448 | MBP | q23 | intron |
| chr18:74702742-74702951 | CpG 23 | q 23 | intergenic |
| chr18:75307909-75308534 | NFATC1 | q23 | intron |
| chr18:75309679-75310023 | NFATC1 | q23 | intron-exon |
| chr18:75311977-75312201 | NFATC1 | q23 | exon-intron |
| chr18:75341420-75341724 | NFATC1 | q23 | intron |
| chr18:75373572-75374591 | NFATC1 | q23 | intron |
| chr18:75410683-75413214 | Cpg 190 | q23 | intergenic |
| chr18:75580087-75580375 | CTDP1 | q23 | intron |
| chr18:75588333-75588535 | CTDP1 | q23 | intron |
| chr18:75686887-75687844 | CpG 74 | q23 | intergenic |
| chr18:8649443-8649672 | CpG 17 | p11.22 | intergenic |
| chr19:10892933-10893195 | CARM1 | p13.2 | intron-exon-intron |
| chr19:11006589-11006790 | SMARCA4 | p13.2 | exon |
| chr19:11150021-11150248 | KANK2 | p13.2 | exon-intron-exon |
| chr19:1319814-1320026 | MUM1 | p13.3 | intron |
| chr19:13849204-13849559 | NANOS3 | p13.12 | exon1 |
| chr19:14134429-14135173 | LPNH1 | p13.12 | exon |
| chr19:17472345-17472664 | SLC27A1 | p13.11 | exon-intron-exon |
| chr19:1926219-1926570 | CSNK1G2 | p13.3 | intron |
| chr19:1949800-1950332 | BTBD2 | p13.3 | intron |
| chr19:19625631-19625873 | ATP13A1 | p13.11 | exon-intron-exon |
| chr19:21457929-21458716 | CpG 56 | p12 | intergenic |
| chr19:2395300-2395522 | LMNB2 | 13.3 | intron-exon |
| chr19:2601659-2601911 | GNG7 | p13.3 | intron |
| chr19:311071-312949 | CpG 183 | p13.3 | intergenic |
| chr19:3483433-3483666 | FZR1 | p13.3 | exon |
| chr19:369898-370191 | SHC2 | p13.3 | exon-intron |
| chr19:40015371-40015792 | LOC400685 | p13.11 | promoter-exon1 |
| chr19:43682103-43682313 | RYR1 | p13.2 | exon |
| chr19:45799137-45799420 | LTBP4 | p13.2 | exon1 |
| chr19:47276356-47276595 | ZNF 574 | p13.2 | exon |
| chr19:4937182-4937405 | KDM4B | p13.3 | intron |
| chr19:5011984-5012242 | KDM4B | p13.3 | intron |
| chr19:5070220-5070445 | KDM4B | p13.3 | intron |
| chr19:5086285-5086545 | KDM4B | p13.3 | intron-exon |
| chr19:53808057-53808453 | FAM83E | p13.33 | Exon1 |
| chr19:53945500-53946033 | FUT1 | p13.3 | exon |
| chr19:5429299-5429577 | CpG 21 | p13.3 | intergenic |
| chr19:55053539-55053793 | PTOV1 | p13.33 | intron-exon-eintron |
| chr19:55518592-55518840 | NCNC3 | p13.33 | exon |
| chr19:5658315-5658524 | LONP1 | p13.3 | intron |
| chr19:5782595-5783297 | FUT6 | p13.3 | exon1 |
| chr19:5794864-5795373 | FUT3 | p13.3 | exon |
| chr19:58486223-58486544 | BIRC8 | q13.41 | intron |
| chr19:5990990-5991228 | RFX2 | p13.3 | exon |
| chr19:61304111-61304555 | ZNF 787 | q13.42 | intron |
| chr19:61824970-61825996 | ZNF71 | q13.43 | exon1 |
| chr19:62972738-62973298 | ZNF 586 | q13.42 | exon1+promoter |
| chr19:7594620-7594832 | XAB2 | p13.2 | exon-intron |
| chr19:7904789-7905410 | TIMM44 | p13.2 | exon-intron-exon-intron |
| chr19:8469441-8470225 | PRAM1 | p13.2 | exon |
| chr2:100304211-100305491 | LORNF2 | q11.2 | exon1 |
| chr2:105219925-105220229 | CpG 29 | q12.1 | intergenic |
| chr2:106325547-106325958 | CpG 33 | q12.2 | intergenic |
| chr2:113524988-113525227 | IL1F8 | q13 | intron |
| chr2:127902567-127902840 | PROC | q14.3 | exon |
| chr2:134665537-134666197 | CpG 57 | q21.2 | intergenic |
| chr2:176657757-176658041 | CpG 21 | q31.3 | intergenic |
| chr2:1821853-1822144 | MYT1L | p25.3 | intron-exon |
| chr2:2168207-2169145 | MYT1L | p25.3 | intron |
| chr2:239698084-239698390 | HDAC4 | q37.3 | intron-exon-intron |
| chr2:241052623-241052867 | GPC1 | q37.3 | intron-exon |
| chr2:37237405-37237906 | EIF2AK2 | p22.2 | promoter |
| chr2:81274715-81276043 | CpG 92 | q12 | intergenic |
| chr2:853930-855091 | CpG 150 | p25.3 | intergenic |
| chr2:91624302-91626790 | CpG 182 | p11.1 | intergenic |
| chr2:97717416-97717689 | ZAP70 | p11.2 | exon-intron |
| chr20:23284868-23285201 | CpG 27 | p11.21 | intergenic |
| chr20:30047981-30048439 | XKR1 | p11.21 | exon |
| chr20:30507601-30507802 | C20orf112 | p11.21 | exon |
| chr20:3157045-3157388 | SLC4A11 | p13 | exon-intron-exon |
| chr20:3157813-3158080 | SLC4A11 | p13 | exon-intron-exon |
| chr20:35464951-35465224 | SRC | q11.23 | exon |
| chr20:390530-391317 | TBC1D20 | q13 | promoter-exon1 |
| chr20:50134672-50135139 | ZFP64 | q13.2 | exon |
| chr20:59882170-59882458 | CDH4 | q13.33 | exon-intron |
| chr20:60403162-60403506 | CABLES2 | q13.33 | exon-intron |
| chr20:60925863-60926192 | COL9A3 | q13.33 | intron-exon |
| chr20:61386379-61386735 | ARFGAP1 | q13.33 | intron-exon |
| chr20:61546504-61546707 | KCNQ2 | q13.33 | exon-intron |
| chr20:61671096-61671397 | PRIC285 | q13.33 | exon |
| chr20:61836693-61836979 | ZGPAT | q13.33 | intron |
| chr20:61891534-61892828 | ZBTB46 | q13.33 | exon-intron |
| chr20:62131405-62131796 | PRPF6 | q13.33 | intron |
| chr21:36181061-36181472 | CpG 42 | q22.12 | intergenic |
| chr21:43350723-43351134 | CBS | q22.3 | intron |
| chr21:43545411-43545640 | CpG 20 | q22.3 | intergenic |
| chr21:44031007-44031479 | CpG 35 | q22.3 | intergenic |
| chr21:44226912-44227270 | AGPAT3 | q22.3 | exon |
| chr21:45234766-45235089 | CpG31 | q22.3 | intergenic |
| chr21:45609558-45609767 | CpG 19 | q22.3 | intergenic |
| chr21:45990214-45991354 | CpG 115 | q22.3 | intergenic |
| chr21:46111440-46111854 | PCBP3 | q22.3 | intron |
| chr21:46633099-46633549 | PCNT | q22.3 | exon-intron |
| chr21:46637679-46637890 | PCNT | q22.3 | intron |
| chr21:9906603-9906958 | CpG 46 | p11.2 | intergenic |
| chr22:21767921-21768431 | GNAZ-RTDR1 | p11.22 | exon |
| chr22:28455352-28455665 | CABP7 | p 12.2 | intron-exon |
| chr22:34150095-34150302 | MCM5 | q12.3 | intron-exon |
| chr22:36575287-36575627 | EIF3L | q13.1 | part of promoter-exon1 |
| chr22:37481403-37482422 | SUN2 | q13.1 | promoter-exon1 |
| chr22:37569114-37570371 | NPTXR | q13.1 | promoter-exon1 |
| chr22:40071877-40072111 | ZC3H7B | q13.2 | intron-exon |
| chr22:41913446-41913774 | CpG 44 | q13.2 | intergenic |
| chr22:45437534-45437778 | GRAMD4 | q13.31 | intron-exon-intron |
| chr22:45448187-45448464 | GRAMD4 | q13.31 | intron-exon-intron |
| chr22:48603161-48603667 | BRD1 | q13.33 | exon |
| chr22:48607339-48607556 | CpG 27 | q13.33 | intergenic |
| chr22:48810340-48811480 | TTLL8 | q13.33 | intron-exon |
| chr22:49068235-49068647 | PLXNB2 | q13.33 | exon-intron-exon |
| chr22:49246827-49247145 | SBF1 | q13.33 | exon-intron-exon |
| chr22:49249915-49250218 | SBF1 | q13.33 | exon-intron-exon |
| chr3:128190305-128191168 | PLXNA1 | q21.13 | exon |
| chr3:131547261-131547527 | COL29A1 | q22.1 | exon |
| chr3:188400102-188400447 | RTP1 | q27.3 | exon |
| chr3:197083418-197083786 | TNK2 | q29 | intron-exon-intron |
| chr3:198869395-198869641 | CpG 19 | q29 | intergenic |
| chr3:20056462-20057430 | KAT2B | p24.3 | part of promoter-exon1 |
| chr3:42675096-42675359 | ZBTB47 | p22.1 | exon |
| chr3:43380423-43380629 | CpG 18 | p22.1 | intergenic |
| chr3:44493842-44494372 | ZNF445 | p21.32 | promoter-exon1 |
| chr3:50357893-50358314 | ZMYND10 | p21.32 | promoter-exon1 |
| chr3:51724794-51725101 | GRM2 | p21.1 | exon |
| chr3:52714577-52715466 | GLT8D1 | p21.1 | devergent promoter |
| chr3:73515426-73516760 | PDZRN3 | p13 | exon |
| chr4:1212291-1212497 | CTBP1 | p16.3 | intron |
| chr4:1323378-1323676 | MAEA | p16.3 | exon |
| chr4:1512396-1512673 | CpG 23 | p16.3 | intergenic |
| chr4:154116501-154116810 | FHDC1 | q31.3 | exon |
| chr4:165328833-165329292 | 1-Mar | q32.3 | intron |
| chr4:25115679-25117174 | CpG 132 | p15.2 | intergenic |
| chr4:2703455-2703818 | FAM 193A | p16.3 | exon |
| chr4:3345629-3345943 | RGS12 | p16.2 | intron |
| chr4:3419448-3419793 | HGFAC | p16.2 | exon-intron-exon |
| chr4:3483246-3483645 | CpG 36 | p.16.2 | intergenic |
| chr4:3489362-3489946 | LRPAP1 | p16.2 | intron-exon-intron |
| chr4:39721906-39722128 | LRPAP1 | p16.2 | exon |
| chr4:519848-520049 | PIGG | p16.3 | intron |
| chr4:56875714-56876904 | KIAA1211 | q12 | exon |
| chr4:77391611-77392084 | FAM47D | q21.1 | promoter |
| chr4:802386-802646 | CPLX1 | p16.3 | intron |
| chr4:860695-860969 | GAK | p16.3 | intron-exon |
| chr4:888030-888532 | GAK | p16.3 | intron-exon |
| chr5:1140533-1140909 | SLC12A7 | p15.33 | intron |
| chr5:114659885-114660201 | CCDC112 | q22.3 | intron-exon |
| chr5:169592408-169592807 | C5orf58 | p35.1 | promoter |
| chr5:169863643-169864049 | KCNIP1 | p35.1 | exon |
| chr5:176696721-176697148 | LMAN | p35.3 | exon-intron-exon |
| chr5:176793468-176793704 | GRK6 | p35.3 | intron-exon |
| chr5:2233251-2233467 | CpG 18 | p15.33 | intergenic |
| chr5:234725-234944 | PLEKHG4B | p15.33 | exon-intron |
| chr5:26705305-26705661 | CpG 28 | p14.1 | intergenic |
| chr5:28845157-28846277 | CpG 76 | p14.1 | intergenic |
| chr5:360286-360592 | AHRR-PDCD6 | p15.33 | intron |
| chr6:109718142-109718923 | CpG 56 | q21 | intergenic |
| chr6:139055256-139056574 | CpG 122 | q23.3 | intergenic |
| chr6:160431926-160432455 | IGF2R | q25.3 | intron |
| chr6:168272831-168273156 | CpG 23 | q27 | intergenic |
| chr6:169390471-169390972 | THBS2 | q27 | exon |
| chr6:25990306-25990539 | CpG 24 | p22.2 | intergenic |
| chr6:35862691-35862892 | C6orf127 | p21.31 | intron-exon |
| chr6:35996457-35997038 | SRPK1 | p21.31 | promoter-exon1 |
| chr6:37037815-37038113 | PI16 | p21.2 | intron |
| chr6:7931332-7931846 | PIP5K1P1 | p24.3 | exon |
| chr6:832605-832908 | CpG 23 | p25.3 | intergenic |
| chr7:105539339-105540384 | SYPL1 | q22.2 | promoter-exon1 |
| chr7:134568859-134569194 | STRA8 | q33 | intron |
| chr7:148825072-148826208 | ZNF 746 | q36.1 | promoter-exon1 |
| chr7:1490067-1490502 | INTS1 | p22.3 | intron-exon-intron |
| chr7:1542262-1542519 | MAFK | p22.3 | intron |
| chr7:156525439-156525722 | CpG 22 | q36.3 | intergenic |
| chr7:156899695-156900109 | DNAJB6 | q36.3 | intron |
| chr7:156901555-156901769 | DNAJB6 | q36.3 | exon |
| chr7:157132785-157135482 | PTPRN2 | q36.3 | intron |
| chr7:157146535-157146969 | PTPRN2 | q36.3 | intron |
| chr7:157270446-157270703 | PTPRN2 | q36.3 | intron |
| chr7:157974134-157974495 | PTPRN2 | q36.3 | intron |
| chr7:158579128-158579356 | VIPR2 | q36.3 | intron |
| chr7:16427303-16427790 | ISPD | p21.1 | promoter-exon1 |
| chr7:16532777-16533199 | CpG 34 | p21.1 | intergenic |
| chr7:1832069-1832294 | MAD1L1 | p21.1 | intron |
| chr7:1857356-1857614 | MAD1L1 | p21.1 | intron |
| chr7:1869288-1869585 | MAD1L1 | p21.1 | intron |
| chr7:1958440-1958648 | MAD1L1 | p21.1 | intron |
| chr7:2011109-2011314 | MAD1L1 | p21.1 | intron |
| chr7:2152297-2152731 | MAD1L1 | p21.1 | intron |
| chr7:2259802-2260102 | CpG 23 | p22.2 | intergenic |
| chr7:2386334-2386866 | EIF3B | p22.2 | exon1 |
| chr7:2532398-2532640 | LFNG | p22.2 | exon-intron |
| chr7:254856-255122 | CpG 29 | p22.3 | intergenic |
| chr7:301534-302448 | FAM20C | p22.3 | intron |
| chr7:4314233-4314543 | CpG 25 | p22.2 | intergenic |
| chr7:4822516-4822738 | RADIL | p22.1 | exon-intron |
| chr7:4823384-4823692 | RADIL | p22.1 | exon-intron |
| chr7:5234274-5234636 | WIP12 | p22.1 | exon-intron |
| chr7:5487894-5488141 | FBXL18 | p22.1 | exon |
| chr7:6176455-6177243 | CYTH3 | p22.1 | intron-exon-intron-exon |
| chr8:1120168-1120390 | CpG 18 | p23.3 | intergenic |
| chr8:11644725-11645178 | GATA4 | p23.1 | intron-exon |
| chr8:1337546-1337984 | CpG 28 | p23.3 | intergenic |
| chr8:1385342-1385928 | CpG 50 | p23.3 | intergenic |
| chr8:141628346-141628550 | EIF2C2 | q24.3 | intron-exon |
| chr8:142300845-142301156 | SLC45A4 | q24.3 | exon-intron |
| chr8:142506215-142506454 | PTP4A3 | q24.3 | exon-intron |
| chr8:143376868-143377185 | TSNARE1 | q24.3 | intron |
| chr8:145183285-145183552 | OPLAH | q24.3 | exon-intron-exon |
| chr8:145632951-145633191 | NFKBIL2 | q24.3 | exon-intron |
| chr8:145784727-145785004 | ARHGAP39 | q24.3 | intron |
| chr8:1767027-1767228 | ARHGEF10 | p23.3 | intron |
| chr8:1818155-1818415 | ARHGEF10 | p23.3 | intron-exon-intron |
| chr8:2034098-2034370 | MYOM2 | p23.3 | exon-intron |
| chr8:21652358-21652615 | GFRA2 | p21.3 | exon |
| chr8:33699906-33700142 | CpG 18 | p12 | intergenic |
| chr8:949722-950154 | CpG 33 | p23.3 | intergenic |
| chr9:111123154-111123370 | CpG20 | q31.3 | intergenic |
| chr9:115900294-115900516 | KIF12 | q32 | intron |
| chr9:129723790-129724191 | PIP5KL1 | q34.11 | exon |
| chr9:131670145-131670644 | USP20 | q34.11 | exon-intron |
| chr9:137296531-137296804 | CpG 24 | q34.3 | intergenic |
| chr9:138106469-138107288 | NACC2 | q34.3 | intron |
| chr9:138513010-138513681 | NOTCH1 | q34.3 | intron-exon-intron-exon |
| chr9:138846038-138846261 | C9orf86 | q34.3 | exon-intron |
| chr9:139832752-139833946 | EHMT1 | q34.3 | intron |
| chr9:21549133-21549816 | LOC554202 | p21.3 | promoter-exon1 |
| chr9:69642237-69643493 | CpG 152 | q12 | intergenic |
| chr9:89629425-89629912 | CpG 40 | q22.1 | intergenic |
| chrX:152191547-152192361 | CpG 57 | q28 | intergenic |
| chrX:153347546-153347968 | PLXNA3 | q28 | exon-intron-exon |
| chrY:106504-107721 | CpG 96 | p11.32 | intergenic |
| chrY:251505-252418 | PPP2R3B | p11.32 | intron |
